# Review of the Neotropical water scavenger beetle genus *Tobochares* Short & García, 2007 (Coleoptera, Hydrophilidae, Acidocerinae): new lineages, new species, and new records

**DOI:** 10.3897/zookeys.1019.59881

**Published:** 2021-02-22

**Authors:** Jennifer C. Girón, Andrew Edward Z. Short

**Affiliations:** 1 Department of Entomology, Purdue University, West Lafayette, IN 47907, USA Purdue University West Lafayette United States of America; 2 Natural Science Research Laboratory, Museum of Texas Tech University, Lubbock, TX 79409, USA Museum of Texas Tech University Lubbock United States of America; 3 Department of Ecology and Evolutionary Biology, and Division of Entomology, Biodiversity Institute, University of Kansas, Lawrence, KS 66045, USA University of Kansas Lawrence United States of America

**Keywords:** Aquatic beetles, seepage habitat, South America, taxonomy, water beetles

## Abstract

The water scavenger beetle genus *Tobochares* Short & García, 2007 currently contains ten species, including one known but formally undescribed taxon. Although *Tobochares* was revised in 2017, ongoing fieldwork as well as an expanded concept of the genus has led to the recognition of numerous additional species. Here a combination of morphological and molecular data is presented to review this newly found *Tobochares* diversity. Fifteen new species are described from South America, bringing the total number of known species to 25: *Tobochares akoerio***sp. nov.** (Suriname), *T. arawak***sp. nov.** (Guyana), *T. anthonyae***sp. nov.** (Venezuela: Bolívar), *T. atures***sp. nov.**, (Venezuela: Amazonas), *T. benettii***sp. nov.** (Brazil: Amazonas), *T. canaima***sp. nov.** (Venezuela: Bolívar), *T. communis***sp. nov.** (Brazil: Amapá and Roraima, Guyana, Suriname, Venezuela: Bolívar), *T. fusus***sp. nov.** (Brazil: Amapá, French Guiana), *T. goias***sp. nov.** (Brazil: Goiás), *T. kappel***sp. nov.** (Suriname), *T. kolokoe***sp. nov.** (Suriname), *T. luteomargo***sp. nov.** (Venezuela: Bolívar), *T. microps***sp. nov.** (Suriname), *T. pemon***sp. nov.** (Venezuela: Bolívar), and *T. romanoae***sp. nov.** (Brazil: Roraima). Both morphological and molecular analyses support four clades within the genus, which are here diagnosed and described as species groups. New distributional records are provided for *T. kusad* Kohlenberg & Short, 2017 and *T. sipaliwini* Short & Kadosoe, 2011, both of which are recorded from Brazil for the first time. Previously restricted to the Guiana Shield region of South America, the distributional range of the genus is now broadly expanded to include localities as far south as the central Brazilian state of Goiás. Consistent with the biology of the previously described species, almost all the new species described here are associated with seepage and wet rock habitats. Remarkably, one species, *T. fusus***sp. nov.**, was collected in both seepage habitats as well as in the rotting fruits of *Clusia* Linnaeus (Clusiaceae), making it one of the few known acidocerines with terrestrial habits outside of the genus *Quadriops* Hansen, 1999. High-resolution images of most species are included, as well as a key to species groups, species, and habitat photographs.

## Abstract (sp)

El género de escarabajos acuáticos detritívoros *Tobochares* Short & García, 2007 actualmente contiene 10 especies, incluyendo un taxón conocido, pero no descrito. Aunque el género fue revisado en 2017, trabajo de campo en curso, así como un concepto ampliado del género han llevado al reconocimiento de numerosas especies adicionales. Aquí presentamos una combinación de datos morfológicos y moleculares para revisar esta diversidad de *Tobochares* recientemente encontrada. Quince especies nuevas son descritas de Suramérica, elevando el número total de especies conocidas a 25: *Tobochares akoerio***sp. nov.** (Surinam), *T. arawak***sp. nov.** (Guyana), *T. anthonyae***sp. nov.** (Venezuela: Bolívar), *T. atures***sp. nov.**, (Venezuela: Amazonas), *T. benettii***sp. nov.** (Brasil: Amazonas), *T. canaima***sp. nov.** (Venezuela: Bolívar), *T. communis***sp. nov.** (Brasil: Amapá y Roraima, Guyana, Surinam, Venezuela: Bolívar), *T. fusus***sp. nov.** (Brasil: Amapá, Guyana Francesa), *T. goias***sp. nov.** (Brasil: Goiás), *T. kappel***sp. nov.** (Surinam), *T. kolokoe***sp. nov.** (Surinam), *T. luteomargo***sp. nov.** (Venezuela: Bolívar), *T. microps***sp. nov.** (Surinam), *T. pemon***sp. nov.** (Venezuela: Bolívar), y *T. romanoae***sp. nov.** (Brasil: Roraima). También encontramos soporte morfológico y molecular para cuatro clados dentro del género, los cuales son aquí diagnosticados y descritos como grupos de especies. Nuevos registros de distribución son provistos para *T. kusad* Kohlenberg & Short, 2017 y *T. sipaliwini* Short & Kadosoe, 2011, ambos registrados para Brasil por primera vez. Previamente restringido a la región del Escudo Guyanés de Suramérica, el rango de distribución del género es ahora ampliamente expandido para incluir localidades tan al sur como el estado de Goiás en el centro de Brasil. Consistentes con la biología de las especies previamente conocidas, casi todas las especies descritas aquí están asociadas con hábitats higropétricos o áreas de drenaje sobre rocas. Notablemente, una especie, *T. fusus***sp. nov.**, fue colectada tanto en hábitats higropétricos como en frutos en descomposición de *Clusia* Linneo (Clusiaceae), constituyéndose en uno de los pocos acidocerinos conocidos con hábitos terrestres fuera del género *Quadriops* Hansen, 1999. Se incluyen imágenes de alta resolución de la mayoría de las especies incluidas, una clave para los grupos de especies y las especies, así como fotografías de los hábitats.

## Introduction

**Figure 1.**
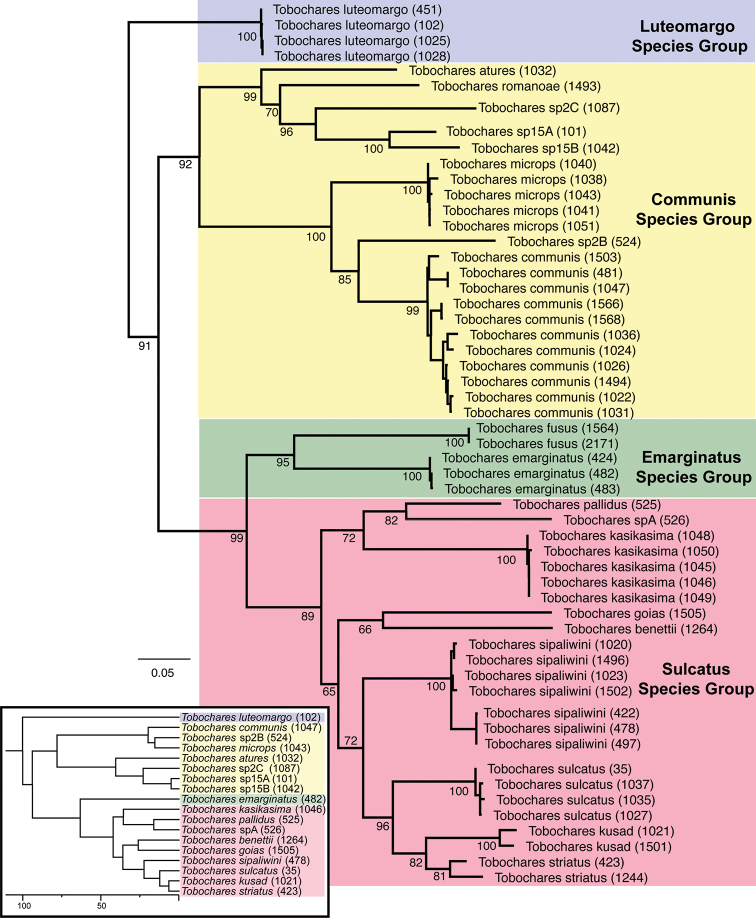
Maximum Likelihood phylogeny of *Tobochares* spp. Inferred from COI sequence data. Numbers next to taxon names are extraction numbers (see Table 1). The *Tobochares* portion of the phylogeny of Acidocerinae based on five gene fragments as presented in Short et al. (2021) is reproduced in the lower left corner for comparative purposes.

**Figure 2.**
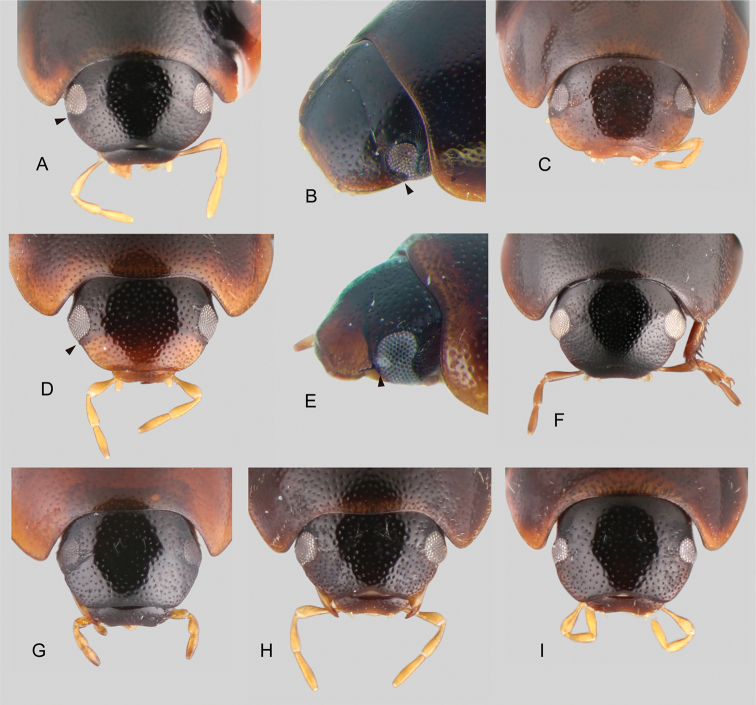
Heads of *Tobochares* spp. **A, B***T. goias*, black mark pointing to canthus emarginating the eye **A** dorsal view **B** anterolateral view **C***T. fusus***D, E***T. luteomargo*, black mark pointing to straight anterior margin of the eye **D** dorsal view **E** anterolateral view **F***T. romanoae***G***T. pemon***H***T. communis***I***T. microps*.

The water scavenger beetles in the genus *Tobochares* Short & García, 2007 are small to minute beetles that are known to occur in seepage and wet rock habitats in northern South America. The genus was just recently revised by Kohlenberg and Short (2017), who recognized ten species, nine of which are described (the tenth species, “sp. A”, is known from a single female and was left undescribed). In the process of doing that revision, they encountered a number of additional species that appeared very similar to *Tobochares*, but also differed in a few notable characters. For example, the anterior margin of the eye is distinctly emarginated by the anterior margin of the frons in all previously described *Tobochares* (e.g., Fig. 2A, B), but not emarginated in these additional species (e.g., Fig. 2D, E). Further, aedeagal forms of these additional species were in some cases quite divergent from those of the described *Tobochares*, such as exhibiting a bifid median lobe (Fig. 11D, E). These morphological differences, combined with very preliminary molecular data that was available to us at that time, made it unclear if these additional taxa were *Tobochares* or belonging to a new genus. A recent comprehensive molecular phylogeny of the Acidocerinae confirmed that these additional taxa (i.e., those with unemarginated eyes) do fall outside the previously delimited boundary of *Tobochares*, but that the generic concept of *Tobochares* could be expanded relatively easily to include them (Short et al. 2021; see inset in Fig. 1). In this contribution, we use a combination of molecular and morphological data to (1) establish four species groups within *Tobochares*, (2) describe fifteen new species, bringing the total number of known species in the genus to 25, and (3) present new records for the previously described species *T. kusad* Kohlenberg & Short, 2017, *T. sipaliwini* Short & Kadosoe, 2011, and *T. striatus* Short, 2013. We present the first record of the genus outside of the Guiana Shield, from the Central Brazilian state of Goiás. Finally, we have confirmed the first terrestrial occurrence of *Tobochares* in rotting *Clusia* fruits and discuss the importance of this new ecological data.

## Materials and methods

### Depositories of examined material

**CBDG**Center for Biological Diversity, University of Guyana, Georgetown;

**INPA**Instituto Nacional de Pesquisas da Amazônia, Manaus, Brazil (N. Hamada);

**MALUZ**Museo de Artrópodos de la Universidad del Zulia, Maracaibo, Venezuela (J. Camacho, M. García);

**MNHN**Muséum national d’Histoire naturelle, Paris, France;

**MIZA** Museo del Instituto de Zoología Agrícola, Maracay, Venezuela (L. Joly);

**NZCS**National Zoological Collection of Suriname, Paramaribo (P. Ouboter, V. Kadosoe);

**SCC** Collection of Simon Clavier, Kourou, French Guiana;

**SEMC** Snow Entomological Collection, University of Kansas, Lawrence, KS (A. Short);

**USNM**U.S. National Museum of Natural History, Smithsonian Institution, Washington, DC (C. Micheli).

### Morphological methods

Slightly more than 1100 specimens were examined. Specimen preparation and examination methods are identical to those given in Girón and Short (2017). Descriptive sequence and morphological terminology follow Kohlenberg and Short (2017). Descriptions of species are grouped by species group and given in alphabetical order, whereas in the habitus figures, species are grouped by similarity for ease of comparison. Figures presented in Kohlenberg and Short (2017) are referred throughout the text to indicate characters already illustrated there. Maps were created using SimpleMappr (Shorthouse 2010). Type labels are cited verbatim in quotation marks.

### Molecular methods

We sequenced the mitochondrial gene COI for eight of the putative 15 new species; we did not have suitable tissue specimens for the remaining species. We also sequenced newly found populations of *T. sipaliwini*, *T. kusad*, and *T. striatus* to further support these species identifications. The number of specimens sequenced per species ranged from one to eleven. All molecular extraction and sequencing methods follow those of Kohlenberg and Short (2017). Resulting DNA sequences were assembled and edited in Geneious R 8.0.5 (Biomatters, http://www.geneious.com/). All sequences are deposited in GenBank (see Table 1 for accession numbers). We combined these newly generated sequences with the COI dataset presented in Kohlenberg and Short (2017) and the *Tobochares* sequences included in Short et al., (2021). IQ-TREE 1.4.4 (Nguyen et al. 2015) was used to infer phylogenetic relationships. The optimal model of substitution was selected using the Auto function in IQ-TREE 1.4.4. In order to assess nodal support, we performed 1000 ultrafast bootstrap replicates (Minh et al. 2013). We included *Crucisternum vanessae* Girón & Short, 2018 as outgroup to root the tree.

## Results

We found morphological support for 15 new species of *Tobochares*, which are described in this contribution. The results of the Maximum Likelihood analysis are presented in Fig. 1. The maximum intraspecific pairwise genetic divergence was 3.9% (*T. communis* sp. nov.). The lowest interspecific pairwise genetic distance between any two species was 7.1% (between *T. kusad* and *T. striatus*). We found potential support for four additional new species (sp. 2B, sp. 2C, sp. 15A, sp. 15B) as indicated in Fig. 1, but these species will await description in a future contribution. In addition, we found support for four reciprocally monophyletic species groups, which we were also able to diagnose morphologically. Although our data are not intended to robustly generate a phylogeny of the genus, the circumscription and phylogenetic relationships we found with this COI data is completely congruent with those recovered by Short et al. (2021), which is based on five genes albeit with far fewer taxa (Fig. 1, insert). Therefore, we believe the general pattern of relationships we recovered to be relatively robust.

**Table 1.** List of specimens and GenBank accession numbers that are used in this study.

Taxon	Extraction	Country: locality	GenBank Accession
*T. atures*	SLE1032	Venezuela: Tobogan de la Selva	MW351439
*T. benettii*	SLE1264	Brazil: nr. Manaus	MW351433
*T. communis*	SLE1024	Venezuela: 15 km NE Pijiguaos	MW349448
	SLE1026	Venezuela: La Escalera	MW349449
	SLE1031	Venezuela: Cuchivero	MW349452
	SLE1036	Venezuela: Pijiguaos	MW349447
	SLE1047	Suriname: Tafelberg Summit	MW351435
	SLE1494	Brazil: Tepequem	MW349450
	SLE1503	Brazil: near Usina de Jatapu	MW349454
	SLE1566	Brazil: nr. Calcoene	MW349455
	SLE1568	Brazil: Oiapoque	MW349456
	SLE481	Suriname: Grensgebergte	MW349453
	SLE1022	Guyana: Kaieteur National Park	MW349451
*T. emarginatus*	SLE424	Suriname: Kasikasima,	KY679835
	SLE482	Suriname: Kasikasima	KY679836
	SLE483	Suriname: Kasikasima	KY679837
*T. fusus*	SLE1564	Brazil: Oiapoque	MW349457
	SLE2171	French Guiana	MW349458
*T. goias*	SLE1505	Brazil: Balneario Lejas	MW351434
*T. kasikasima*	SLE1045	Suriname: Kappel Airstrip	KY679850
	SLE1046	Suriname: Kappel Airstrip	KY679851
	SLE1048	Suriname: Tafelberg Summit	KY679849
	SLE1049	Suriname: Tafelberg Summit	KY679852
	SLE1050	Suriname: Tafelberg Summit	KY679848
*T. kusad*	SLE1021	Guyana: Kusad Mts.	KY679846
	SLE1501	Brazil: near Usina de Jatapu	MW349441
*T. luteomargo*	SLE102	Venezuela: Cuchivero	KC935283
	SLE1025	Venezuela: 15 km NE Pijiguaos	MW349461
	SLE1028	Venezuela: Pijiguaos	MW349462
	SLE451	Venezulea: Campamento Río Aro	MW349460
*T. microps*	SLE1038	Suriname: Tafelberg Summit	MW349443
	SLE1040	Suriname: Tafelberg Summit	MW349444
	SLE1041	Suriname: Tafelberg Summit	MW349445
	SLE1043	Suriname: Tafelberg Summit	MW351436
	SLE1051	Suriname: Tafelberg Summit	MW349446
*T. pallidus*	SLE525	Venezuela: Tobogan de la Selva	KY679853
*T. romanoae*	SLE1493	Brazil: Tepequem	MW349459
*T. sipaliwini*	SLE1020	Guyana: Kusad Mts.	KY679841
	SLE1023	Suriname: Kwamala	KY679842
	SLE422	Suriname: Kasikasima	KY679838
	SLE478	Suriname: Kasikasima	KY679839
	SLE497	Suriname: Kasikasima	KY679840
*T. striatus*	SLE1244	Suriname: Sipaliwini Savanna	MW349442
	SLE423	Suriname: Kasikasima	KY679847
*T. sulcatus*	SLE0035	Venezuela: Tobogan de la Selva	KC935327
	SLE1027	Venezuela: Tobogan de la Selva	KY679845
	SLE1035	Venezuela: Tobogan de la Selva	KY679843
	SLE1037	Venezuela: Pijiguaos	KY679844
*T.* sp. *A*	SLE526	Venezuela: Tobogan de la Selva	KY679854

### Taxonomy

#### Genus Tobochares

Taxon classificationAnimaliaColeopteraHydrophilidae

Short & García, 2007: 2

A4BFA9EF-222B-5469-81AA-C59ABEEF5B18

##### Type species.

*Tobochares sulcatus* Short & García, 2007: 4; by original designation.

##### Differential diagnosis.

**Figure 3.**
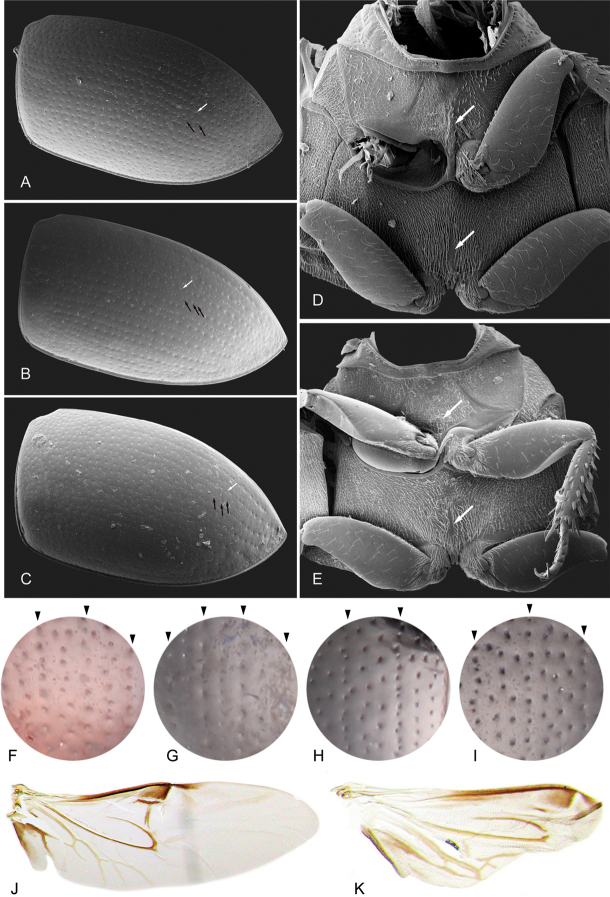
Characters of elytra and wings of *Tobochares* spp. **A–E** Scanning Electron Micrographs **A–C** elytra: white arrows point to serial punctures; black arrows point to interserial punctures **A***T. communis***B***T. atures***C***T. arawak***D, E** ventral view of mesoventrite: **D***T. communis*: top white arrow points to posterior elevation of mesoventrite with low longitudinal elevation; bottom white arrow points to narrow longitudinal glabrous patch of the metaventrite **E***T. arawak*: top white arrow points to posterior elevation of mesoventrite with weakly elevated bulge; bottom white arrow points to narrow longitudinal glabrous patch of the metaventrite. **F–I** detail of elytral punctation; black marks at top of each circle indicates serial punctures **F***T. pemon* (all punctures relatively large; serial punctures longitudinally aligned; interserial punctures in irregular single row) **G***T. atures* (all punctures longitudinally aligned; serial punctures larger than interserial punctures; interserial punctures more densely distributed) **H***T. kolokoe* (serial punctures longitudinally aligned; interserial punctures forming one or two irregular rows) **I***T. canaima* (serial punctures longitudinally aligned; interserial punctures forming two or three irregular rows) **J, K** hindwings **J***T. sipaliwini***K***T. microps*.

Small beetles, total body length 1.5–2.6 mm. Color yellowish brown, orange brown to dark brown. Body shape oval in dorsal view; moderately (Fig. 5B) to strongly convex in lateral view (e.g., Fig. 9E). Antennae with eight antennomeres (e.g., Kohlenberg and Short 2017: fig. 8). Maxillary palps curved inward, short (e.g., slightly shorter than the width of the head) and slender (Fig. 2A; e.g., *T. goias* sp. nov.), to very short (nearly half the width of the head) and stout (Fig. 2G; e.g., *T. pemon* sp. nov.). Elytra without sutural striae, but in *T. akoerio* sp. nov. and *T. romanoae* sp. nov. stria I more strongly impressed along posterior half of elytra (Fig. 8A and D, respectively); elytral punctures seemingly arranged in rows, in some species more evidently so (Fig. 8); in some species interserial punctures not longitudinally aligned (Figs 9, 10); serial punctures sometimes impressed into distinct grooves (e.g., Kohlenberg and Short 2017: fig. 2). Prosternum flat (e.g., Fig. 5C). Posterior elevation of mesoventrite either flat, bulging (Fig. 3E) or with a transverse or longitudinal ridge (Fig. 3D). Metaventrite densely pubescent, except for a median glabrous patch, which is either ovoid and wide (e.g., Kohlenberg and Short 2017: fig. 10) or longitudinal and narrow (Fig. 3D, E). Posterior femora mostly glabrous, with only few scattered setae, sometimes with hydrofuge pubescence along basal half of anterodorsal margin (Kohlenberg and Short 2017: fig. 22). Fifth abdominal ventrite apically evenly rounded, without stout setae (e.g., Kohlenberg and Short 2017: fig. 13). Aedeagus with basal piece usually very short (nearly one third of the length of parameres; Fig. 11).

###### Remarks on diagnostic features of *Tobochares* Short & García, 2007

***Body shape and coloration*.** In lateral view, the degree of convexity of the body can be diagnostic, as only a few species (all in the *communis* species group) are comparatively strongly convex (e.g., *T. kolokoe* sp. nov., Fig. 9E; *T. akoerio*, Fig. 8E). Except for a few cases (e.g., the paler elytral margins of *T. luteomargo* sp. nov., Fig. 5B) the general dorsal coloration of the body in *Tobochares* is not particularly useful for diagnosis. The coloration of the head is diagnostic for some species of the *sulcatus* species group.

***Maxillary palps*.** In general, the maxillary palps in *Tobochares* are short (nearly as long as the width of the head; e.g., Fig. 2A, D, F, H, I) or very short (nearly half as long as the width of the head; e.g., Fig. 2G). The longer palps are slender, as the regular acidocerine maxillary palps (e.g., *Crucisternum* Girón & Short, 2018), but in the shorter forms, the maxillary palps are also stout: each palpomere is shorter and apically wider (or mesally in the case of maxillary palpomere IV). The coloration of palpomere IV (uniformly yellowish vs. apically darkened) can be diagnostic for some species, especially in the *sulcatus* species group (see Kohlenberg and Short 2017).

***Eyes*.** The direction of the anterior margin of the eye in dorsal view is partly diagnostic at the species-group level. The anterior margin of the eye is oblique and posteriorly directed in the *sulcatus* and *emarginatus* species groups (Fig. 2A–C), forming a reduced to well-developed canthus, which in lateral view of the head results in a clearly emarginate anterior margin of the eye (Fig. 2B); it is oblique and anteriorly directed to perpendicular to the outer margin of the head in the *luteomargo* and *communis* species groups (Fig. 2D–I), which in lateral view of the head results in a straight anterior margin of the eye (Fig. 2E), only rarely slightly emarginated. The outer margin of the eyes can also be considered diagnostic, being continuous with the outline of the head (eyes not bulging) in the *sulcatus* species group (Fig. 2A), or slightly bulging from the outline of the head in the *luteomargo* and *communis* species groups (e.g., Fig. 2D, F).

***Mesoventrite*.** The posterior elevation of the mesoventrite exhibits high variation within *Tobochares*. It usually bears a low, transverse medial ridge, but it can also be flat, or with a weakly elevated bulge (Fig. 3E), or a broad and low longitudinal ridge (Fig. 3D). Not necessarily a diagnostic feature at the species-group level.

***Metaventrite*.** The surface of the metaventrite is overall densely covered by hydrofuge pubescence, with a posteromedial glabrous patch. The shape of the glabrous patch is diagnostic at the species-group level: members of the *sulcatus* species group have a broad, ovoid to diamond-shaped glabrous patch (Kohlenberg and Short 2017: fig. 10), whereas in the *luteomargo* and *communis* species groups, the glabrous patch is shaped like a narrow longitudinal bar (Fig. 3D, E).

***Elytral punctation*.** The distribution and characteristics of the elytral punctation are highly variable in *Tobochares*. The disposition and degree of impression of the elytral punctation (i.e., serial punctures, ground punctures and systematic punctures) are useful for species recognition. The elytral punctures are generally aligned in rows, but this is not so evident in some species (e.g., *T. luteomargo* (Fig. 5A), *T. communis* (Figs 3A, 6A), *T. microps* sp. nov. (Fig. 6D), *T. fusus* sp. nov. (Fig. 5D)), where the punctures seem to be evenly distributed rather than longitudinally aligned. In species of the *luteomargo* and *emarginatus* species groups, and some species in the *communis* species group all the elytral punctures are similar in size and degree of impression. Most members of the *sulcatus* species group have well developed, impressed, elytral striae (see Kohlenberg and Short 2017). The sutural striae are always absent in *Tobochares*, but in *T. akoerio* (Fig. 8A) and *T. romanoae* (Fig. 8D) the impressed stria I on each elytron is more strongly impressed along the posterior half of the elytra, which might resemble a well-developed sutural stria. In some species the elytral striae are only impressed along the posterior half or third of the elytra (e.g., *T. kasikasima* Short, 2013, Kohlenberg and Short 2017: fig. 3C; *T. romanoae* (Fig. 8D) and *T. akoerio* (Fig. 8A)).

In some species in the *communis* species group, the serial punctures can be recognized by their higher density in comparison with interserial punctures (Figs 3H, I, 10A, D), whereas in others the serial punctures are larger and less dense than the interserial punctures (*T. atures* sp. nov., Figs 3B, G, 7D). Seta bearing systematic punctures are distributed in irregular rows in the *sulcatus* species group, whereas in the *luteomargo* and *communis* species groups, seta bearing systematic punctures are rather scarce, sometimes only evident along the sides and posterior third of elytra (e.g., Fig. 3A–C). Some details of the elytral punctation require high magnification to be properly observed.

***Hind wings*.** Most species of *Tobochares* have well-developed hind wings (Fig. 3J), with the notable exception of *T. microps*, which is polymorphic, with individuals exhibiting either full size or brachypterous wing forms (e.g., Fig. 3K).

***Metafemora*.** For the most part, the anterior surface of the metafemora is glabrous, smooth and shiny, with only few scattered setae and very shallow reticulations (Kohlenberg and Short 2017: fig. 12). Sometimes there is a narrow strip of hydrofuge pubescence along the basal half of the anterodorsal margin. Not necessarily a diagnostic feature at the species-group level.

***Abdomen*.** All the abdominal ventrites are uniformly covered by fine pubescence, which varies in density: in *T. canthus* Kohlenberg & Short, 2017, *T. emarginatus* Kohlenberg & Short, 2017, and *T. luteomargo* the pubescence is rather scanty, whereas in the remainder species of the genus the pubescence is very dense (Kohlenberg and Short 2017: fig. 13). The posterior margin of the fifth ventrite is evenly rounded and lacks thick, flat spine-like setae (Kohlenberg and Short 2017: fig. 13).

***Aedeagus*.** As is usual in acidocerines, the configuration of the aedeagus is diagnostic at the species and species-group level, although, it can be considered highly variable within the genus, which is unusual in the subfamily. The basal piece is usually short (between 0.3 and 0.6 × the length of the parameres), except in *T. luteomargo*, in which the basal piece is slightly longer than the parameres (Fig. 11D). The median lobe varies from roughly triangular and apically rounded (as in most species of the *sulcatus* species group, Kohlenberg and Short 2017: fig. 14) to rather sagittate, either narrowing along its apical third (e.g., Fig. 11G–I), or apically pinched (e.g., Fig. 11K–M). The apex of the median lobe is rarely emarginate at apex (e.g., Fig. 11D, E). The parameres range in length from shorter to nearly as long as the median lobe, with outer margins straight, broadly curved or sinuate. The overall shape and proportions of the aedeagus, particularly the median lobe, are variable in *Tobochares*.

### Key to the species groups of *Tobochares*

**Table:** 

1	Anterior margin of eye emarginate in lateral view, oblique and posteriorly directed in dorsal view (Fig. 2A–C); glabrous patch on metaventrite broad, ovoid to diamond-shaped (Kohlenberg and Short 2017: fig. 10)	**2**
–	Anterior margin of eye straight, at most only slightly emarginate in lateral view, oblique and anteriorly directed in dorsal view (Fig. 2D–I); glabrous patch on metaventrite shaped as a narrow longitudinal bar (Fig. 3D, E)	**3**
2	In lateral view, eye narrowing to about a quarter of its dorsal width	***emarginatus* species group**
–	In lateral view, eye narrowing to about half or slightly less of its dorsal width	***sulcatus* species group**
3	Hydrofuge pubescence on abdominal ventrites scanty; few metatibial spines, reduced in size; metatarsomere 2 much shorter than metatarsomere 5	***luteomargo* species group**
–	Hydrofuge pubescence on abdominal ventrites very densely distributed; metatibial spines large and rather dense; metatarsomere 2 nearly as long as metatarsomere 5	***communis* species group**

The complete list of species including their assigned species group and known distribution are recorded in Table 2.

**Table 2.** Checklist of *Tobochares* species, their assigned species group, and known distribution. Asterisks (*) denote new country records for previously described species.

Species group	Species	Known distribution
* communis *	*Tobochares akoerio* sp. nov.	Suriname
	*Tobochares anthonyae* sp. nov.	Venezuela (Bolívar)
	*Tobochares arawak* sp. nov.	Guyana
	*Tobochares atures* sp. nov.	Venezuela (Bolívar)
	*Tobochares canaima* sp. nov.	Venezuela (Amazonas)
	*Tobochares communis* sp. nov.	Brazil (Amapá, Roraima), Guyana, Suriname, Venezuela (Bolívar)
	*Tobochares kappel* sp. nov.	Suriname
	*Tobochares kolokoe* sp. nov.	Suriname
	*Tobochares microps* sp. nov.	Suriname
	*Tobochares pemon* sp. nov.	Suriname
	*Tobochares romanoae* sp. nov.	Brazil (Roraima)
* emarginatus *	*Tobochares canthus* Kohlenberg & Short, 2017	Venezuela (Amazonas)
	*Tobochares emarginatus* Kohlenberg & Short, 2017	Suriname
	*Tobochares fusus* sp. nov.	Brazil (Amapá), French Guiana
* luteomargo *	*Tobochares luteomargo* sp. nov.	Venezuela (Bolívar)
* sulcatus *	*Tobochares benettii* sp. nov.	Brazil (Amazonas)
	*Tobochares canaliculatus* Kohlenberg & Short, 2017	Venezuela (Amazonas)
	*Tobochares goias* sp. nov.	Brazil (Goiás)
	*Tobochares kasikasima* Short, 2013	Suriname
	*Tobochares kusad* Kohlenberg & Short, 2017	Guyana, Brazil* (Roraima)
	*Tobochares pallidus* Kohlenberg & Short, 2017	Venezuela (Amazonas, Bolívar)
	*Tobochares sipaliwini* Short & Kadosoe, 2011	Suriname, Brazil* (Roraima)
	*Tobochares* sp. A in Kohlenberg & Short, 2017	Venezuela (Amazonas)
	*Tobochares striatus* Short, 2013	Suriname
	*Tobochares sulcatus* Short & García, 2007	Venezuela (Amazonas, Bolívar)

#### Tobochares sulcatus

Taxon classificationAnimaliaColeopteraHydrophilidae

species group

C005666E-376B-50FC-8C4C-154284A560E4

##### Diagnosis.

This species group can be recognized by the oblique and posteriorly directed anterior margin of the eye in lateral view, which emarginates the eye in lateral view (Fig. 2A–C), the broad, ovoid to diamond-shaped glabrous patch on the metaventrite (Kohlenberg and Short 2017: fig. 10), the dense and uniform distribution of the hydrofuge pubescence on the abdominal ventrites (Kohlenberg and Short 2017: fig. 13A), and the moderate (in number and size) metatibial spines.

##### Composition.

This species group includes the following species: *Tobochares benettii* sp. nov., *T. canaliculatus* Kohlenberg & Short, 2017, *T. goias* sp. nov., *T. kasikasima* Short, 2013, *T. kusad* Kohlenberg & Short, 2017, *T. pallidus* Kohlenberg & Short, 2017, *T. sipaliwini* Short & Kadosoe, 2011, *T. striatus* Short, 2013, and *T. sulcatus* Short & García, 2007; see Table 2.

### Key to the species of the *Tobochares sulcatus* species group

Modified from Kohlenberg and Short (2017)

**Table:** 

1	Elytra with impressed grooves along their entire length (e.g., Kohlenberg and Short 2017: fig. 11C, E, F)	**2**
–	Elytra with impressed grooves only along posterior half or less, or completely without grooves (e.g., Kohlenberg and Short 2017: fig. 11A, B, D)	**5**
2	Apical maxillary palpomere uniformly pale (Kohlenberg and Short 2017: fig. 8D). Pronotum and elytra light brown to brown, head brown, clypeus with large, distinctly pale preocular patches (Kohlenberg and Short 2017: fig. 4A) (Venezuela)	***Tobochares canaliculatus* Kohlenberg & Short**
–	Apical maxillary palpomere darkened at least at apex, and sometimes on distal half or more (Kohlenberg and Short 2017: fig. 8A–C). Pronotum and elytra brown to dark brown, head dark brown to black, clypeus with small, pale preocular patches	**3**
3	Punctures within elytral grooves small, grooves appearing fairly smooth (Kohlenberg and Short 2017: fig. 11F). Elevation of mesoventrite forming transverse carina without tooth, not elevated to same plane as the ventral surface of the mesocoxae (Venezuela)	***Tobochares sulcatus* Short & García**
–	Punctures within elytral grooves strongly impressed and distinct (Kohlenberg and Short 2017: fig. 11C). Elevation of mesoventrite forming transverse carina with tooth, elevated to same plane as the ventral surface of the mesocoxae	**4**
4	Apical maxillary palpomere with apex ranging from slightly to distinctly darkened (Kohlenberg and Short 2017: fig. 8A). Eyes emarginate at lateral margin, narrowing to roughly two thirds the width (Kohlenberg and Short 2017: fig. 5A, B) (Brazil: Roraima, Guyana)	***Tobochares kusad* Kohlenberg & Short**
–	Apical maxillary palpomere darkened in at least distal half (Kohlenberg and Short 2017: fig. 8B). Eyes emarginate at lateral margin, narrowing to slightly greater than half the width (Kohlenberg and Short 2017: fig. 5C, D) (Suriname)	***Tobochares striatus* Short**
5	Elytra with grooves visible along posterior two thirds or less, grooves most prominent near elytral suture (e.g., Kohlenberg and Short 2017: fig. 11A, B), or at least with serial punctures clearly aligned longitudinally	**6**
–	Elytra without any trace of grooves along their entire length; all elytral punctures seemingly uniformly distributed, not forming clear longitudinal rows	**9**
6	General coloration dark; elytral grooves visible along posterior two thirds of elytra	**7**
–	General coloration pale; if present, elytral grooves only visible along posterior quarter of elytra	**8**
7	Elytra with grooves on posterior half (Kohlenberg and Short 2017: fig. 11A). Apical maxillary palpomere uniformly pale (Kohlenberg and Short 2017: fig. 7A). Posterior elevation of mesoventrite forming a medially prominent and acute transverse carina (Kohlenberg and Short 2017: fig. 9E) (Brazil: Roraima, Guyana, Suriname)	***Tobochares sipaliwini* Short & Kadosoe**
–	Elytra with grooves on posterior third (Kohlenberg and Short 2017: fig. 11B). Apical maxillary palpomere with apex darkened (Kohlenberg and Short 2017: fig. 7B). Posterior elevation of mesoventrite forming a medially prominent and blunt transverse carina (Kohlenberg and Short 2017: fig. 9D) (Suriname)	***Tobochares kasikasima* Short**
8	Elytra without grooves, but with serial punctures clearly aligned longitudinally (Kohlenberg and Short 2017: fig. 11D). Eyes emarginate at lateral margin, narrowing to about half of the width (Kohlenberg and Short 2017: fig. 6A, B) (Venezuela)	***Tobochares pallidus* Kohlenberg & Short**
–	Elytra with weak grooves on posterior quarter. Eyes emarginate at lateral margin, narrowing to slightly less than half of the width (Venezuela)	***Tobochares* sp. A (sensu Kohlenberg and Short 2017**)
9	General coloration uniform orange brown along pronotum and elytra, with dark brown head. Posterior elevation of mesoventrite forming a medially prominent (acute) curved transverse ridge (Brazil: Amazonas)	***Tobochares benettii* sp. nov.**
–	General coloration dark brown with paler (yellowish to orange) lateral margins of pronotum and elytra. Posterior elevation of mesoventrite forming a low and uniform curved transverse ridge (Brazil: Goiás)	***Tobochares goias* sp. nov.**

#### Tobochares benettii sp. nov.

Taxon classificationAnimaliaColeopteraHydrophilidae

8D05A782-189D-587D-8060-4D432D27B950

http://zoobank.org/A8163D90-1FF7-4273-854A-BDBB330DBB0E

**Figure 4.**
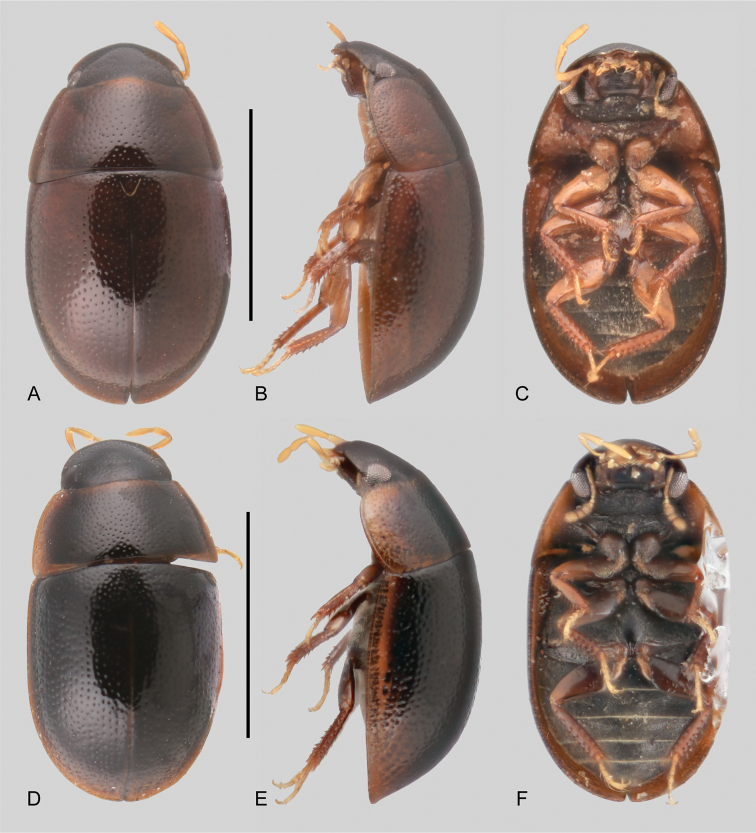
Habitus of *Tobochares* spp. in the sulcatus species group **A–C***T. benettii***A** dorsal view **B** lateral view **C** ventral view **D–F***T. goias***D** dorsal view **E** lateral view **F** ventral view. Scale bars: 1 mm.

Figs 4A–C , 11A , 13 , 14A, B

Tobochares sp. B: Short et al. (2021).

##### Type material examined.

***Holotype* (male)**: “Brazil: Amazonas: Rio Preto da Eva; -2.678466, -59.401714, 25 m; ca. 32 Km W of Rio Preto da Eva; seepage on sandstone with algae; 10.vi.2017; leg. Benetti and team; BR17-0610-01A” (INPA). ***Paratypes* (8 exs.): Brazil: Amazonas**: Same data as holotype (8, INPA, SEMC including DNA voucher SLE1264).

##### Differential diagnosis.

*Tobochares benettii* can be easily recognized from all other *Tobochares* species in the *sulcatus* species group by its elytral punctures seemingly uniformly distributed, not forming clear longitudinal rows, therefore completely lacking elytral striae (Fig. 4A). This character makes it similar to *Tobochares goias*, from which it can be distinguished by its uniform orange brown coloration along pronotum and elytra, with dark brown head (Fig. 4A–C), the posterior elevation of the mesoventrite forming a curved transverse ridge, which is medially prominent and acute and by characters of the aedeagus (Fig. 11A).

##### Description.

***Size and form***: Body length 1.6–2.0 mm. Body elongate oval, strongly convex (Fig. 4A, B). ***Color and punctation*.** Head dark brown, pronotum and elytra uniformly orange brown; antennae, mouthparts and legs orange to yellowish brown, with paler tarsi (Fig. 4C). Ground punctation on head, pronotum and elytra moderately marked. ***Head***: Eyes in dorsal view with anterior margin oblique, posteriorly directed, and outer margins continuous with outline of head (as in Fig. 2A); in lateral view, eyes emarginate to about half the length of eye (as in Fig. 2B). Maxillary palps slender, nearly as long as the width of the head, uniformly yellow in color (as in Fig. 2A). ***Thorax***: Elytra with slightly defined rows of punctures, not forming grooves (Fig. 4A). Elevation of mesoventrite forming a somewhat transverse bulge (Fig. 4C). Metaventrite with distinct median, broad, ovoid glabrous area extending along posterior two thirds (Fig. 4C). ***Abdomen***: Abdominal ventrites uniformly and very densely pubescent. Aedeagus (Fig. 11A) with basal piece 0.25 × the length of a paramere; widest point of parameres (near base) nearly 2/3 greatest width of median lobe (near base), with outer margins slightly sinuate, and rounded apex; median lobe roughly triangular, dorsally concave, apical region nearly half as wide as base, broadly rounded at apex; gonopore situated at apex of median lobe.

##### Etymology.

Named after Cesar J. Benetti, Brazilian specialist on aquatic beetles, in honor of his contributions to Neotropical beetle taxonomy and for all his assistance in the field.

##### Distribution.

Only known from the type locality in Amazonas State, Brazil, situated slightly north of the Amazon River (Fig. 13).

##### Life history.

This only known series was collected on a vertical seepage on sedimentary rock (Fig. 14A, B).

#### Tobochares goias sp. nov.

Taxon classificationAnimaliaColeopteraHydrophilidae

BD22AB85-DE46-525E-A309-E7B921CBCFAA

http://zoobank.org/E4629680-E024-4DC5-BDF1-A85D8A3995DE

Figs 2A, B , 4D–F , 11B , 12 , 15C

Tobochares sp. C: Short et al. (2021)

##### Type material examined.

***Holotype* (male)**: “Brazil: Goiás: Cristalina: -16.87004, -47.61716; 947 m; Cristalina Balneario Lajes; seepage on rock next to river; 4.iii.2018; Benetti and team; BR18-0304-02B.” (INPA). ***Paratypes* (26 exs.): Brazil: Goiás**: Same data as holotype (26, INPA, SEMC including DNA Voucher SLE1505).

##### Differential diagnosis.

*Tobochares goias* can be easily recognized from most species in the *sulcatus* species group by its elytral punctures seemingly uniformly distributed, not forming clear longitudinal rows, therefore completely lacking elytral striae (Fig. 4D). This character makes it similar to *T. benettii*, from which it can be distinguished by its dark brown coloration, with paler (yellowish to orange) lateral margins of pronotum and elytra (Fig. 4D, E), the posterior elevation of the mesoventrite forming a low and uniform curved transverse ridge (Fig. 4F), and by characters of the aedeagus (Fig. 11B).

##### Description.

Dorsal surfaces of body dark brown with paler outer margins of pronotum and elytra (Fig. 4D, E); head slightly darker; mouthparts and antennae yellowish; legs yellowish to brown with paler tarsi (Fig. 4F). Ground punctation on head, pronotum and elytra moderately marked. ***Head***: Eyes in dorsal view with anterior margin oblique, posteriorly directed, and outer margins continuous with outline of head; in lateral view, eyes emarginate to about half the length of eye (Fig. 2A, B). Maxillary palps slender, slightly shorter than width of head, uniformly yellowish in color (Fig. 2A). ***Thorax***: Elytra with slightly defined rows of punctures, not forming grooves (Fig. 4D). Elevation of mesoventrite with a low transverse ridge (Fig. 4F). Metaventrite with distinct median, broad, ovoid glabrous area extending along posterior two thirds (Fig. 4F). ***Abdomen***: Abdominal ventrites uniformly and very densely pubescent. Aedeagus (Fig. 11B) with basal piece 0.2 × the length of a paramere; widest point of parameres (near base) nearly as wide as basal width of median lobe, with outer margins very slightly sinuate, and rounded apex; median lobe fusiform, with widest point slightly apicad of midpoint, broadly rounded at apex; gonopore situated at apex of median lobe.

##### Etymology.

Named after the Brazilian state of Goiás, from which the species is known.

##### Distribution.

Only known from the type locality in the central Brazilian state of Goiás. This is the first and currently only species of *Tobochares* reported from south of the Amazon River (Fig. 12).

##### Life history.

This species was collected on wet rock along the margins of the Ribeirão das Lejas. See Fig. 14C.

### New records from Brazil and Suriname

#### Tobochares kusad

Taxon classificationAnimaliaColeopteraHydrophilidae

Kohlenberg & Short, 2017

B8A4BDC0-2CC7-58F4-9A78-635F7623A4C4

Figs 12 , 14D

##### New material examined.

**Brazil: Roraima**: Caroebe Municipality, Reservoir by Usina de Jatapú, 0.872953°, -59.282170°, 185 m, 17.i.2018, large wall seep with algae, leg. Short, Benetti, and Santana, BR18-0117-01A (7, INPA, SEMC including DNA Voucher SLE1501).

#### Tobochares sipaliwini

Taxon classificationAnimaliaColeopteraHydrophilidae

Short & Kadosoe, 2011

93E066E5-CD5D-5316-A45C-C5D4F1BEE31C

Fig. 12 , 14E

##### New material examined.

**Brazil: Roraima**: Amajari Municipality, Serra do Tepequém, Igarape Preto Negro, Cachoeira Laje Preta, 3°36.381'N, 61°42.878'W, 618 m, 14.i.2018, leg. Short and Benetti, BR18-0114-04B (12, INPA, SEMC, including DNA voucher SLE1496); Caroebe Municipality, Reservoir by Usina de Jatapú, 0.872953, -59.282170, 185 m, 17.i.2018, large wall seep with algae, leg. Short, Benetti, and Santana, BR18-0117-01A (1, SEMC including DNA Voucher SLE1502).

#### Tobochares striatus

Taxon classificationAnimaliaColeopteraHydrophilidae

Short, 2013

D91EC0BF-B8FD-5C50-9475-FEC68807C861

Fig. 12

##### New material examined.

**Suriname: Sipaliwini**: Sipaliwini Savanna Nature Reserve, 2°00.240'N, 55°58.259'W, 374 m, 4-Brothers Mountains, leg. Short and Baca, 30.iii.2017, seeps on granite SR17-0330-04A (1, SEMC including DNA Voucher SLE1244).

#### Tobochares luteomargo

Taxon classificationAnimaliaColeopteraHydrophilidae

species group

B9E3BC41-8489-5B7C-BC48-6CA8F4B9F637

##### Diagnosis.

**Figure 5.**
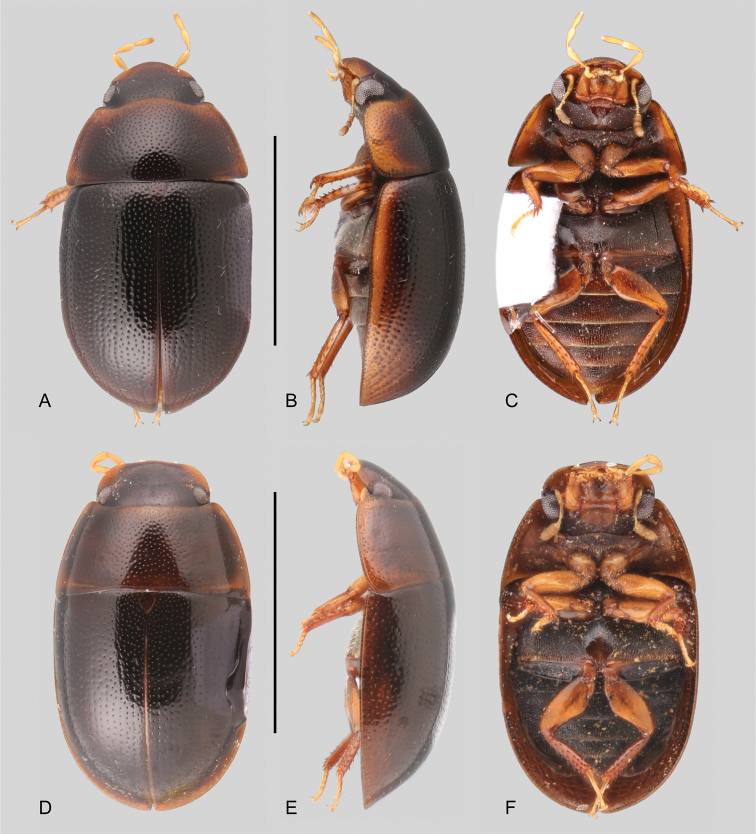
Habitus of *Tobochares* spp. in the *luteomargo* and *emarginatus* species group **A–C***T. luteomargo***A** dorsal view **B** lateral view **C** ventral view. **D–F***T. fusus***D** dorsal view **E** lateral view **F** ventral view. Scale bars: 1 mm.

Members of this species group can be recognized by the straight anterior margin of the eye in lateral view (Fig. 2E), the longitudinally aligned and undifferentiated elytral punctures with about the same size and degree of impression (Fig. 5A), the short and narrow glabrous patch on the metaventrite (Fig. 5C), the scantiness of the hydrofuge pubescence on the abdominal ventrites (Fig. 5C), and the reduced (in number and size) metatibial spines.

##### Composition.

This species group currently contains only a single species, *T. luteomargo* sp. nov.

#### Tobochares luteomargo sp. nov.

Taxon classificationAnimaliaColeopteraHydrophilidae

96950BE2-9DDC-521F-A024-F6DA894AE37B

http://zoobank.org/E6802D72-6F89-44EB-BCEC-D09249FD19BE

Figs 2D, E , 5A–C , 11D , 13 , 15C, D

Tobochares sp. 10: Short et al. (2021).

##### Type material examined.

***Holotype* (male)**: “Venezuela: Bolivar State/ 7°41'23.6"N, 64°1'56.0"W, 134 m/ ca. 14 km E Rio Aro; 5.viii.2018/ leg. A. Short and M. García/ AS-08-073; rock outcropping” (MIZA). ***Paratypes* (282 exs.): Venezuela: Bolívar**: “6°35.671'N, 66°49.238'W; 80 m; Los Pijiguaos; morichal/ rock outcrop; 16.ix.2007; leg. Short, García, Joly; AS-07-015” (17, SEMC including DNA vouchers SLE1028); same, except “seeps and stream at night; 9.vii.2010; leg. Short and Téllez; VZ10-0709-03A” (1, SEMC); Same data as holotype (80, MALUZ, SEMC); “7°29'47.3"N, 65°51'44.8"W; 45 m; 2 Km E of Río Cuchivero; rock outcrop seeps; 6.viii.2008; leg. Short, Téllez, Arias; AS-08-075” (3, SEMC); “6°57.904'N, 66°36.392'W; 51 m; outcrop ca. 15 km NE of Los Pijiguaos; detritus flotation; 9.vii.2010; leg. Short and Téllez; VZ10-0709-01B” (2, SEMC including DNA voucher SLE1025); “7°27.501'N, 65°52.093'W; 45 m; Rock outcrop by Río Cuchivero; seeps; 10.vii.2010; leg. Short, Téllez, Arias; VZ10-0710-01A” (75, SEMC including DNA voucher SLE102); “7°37.443'N, 64°08.324'W; 90 m; Campamento Río Aro, by Río Aro; rock pools; 11.vii.2010; leg. Short, Téllez, Arias; VZ10-0711-01A” (2, SEMC); same, except “seep scrubbing; VZ10-0711-01B” (48, SEMC); same, except “seep/rock pools at night; VZ10-0711-01E” (44, SEMC including DNA voucher SLE451).

##### Differential diagnosis.

*Tobochares luteomargo* can be distinguished by the yellow band along the outer margins of the body (Fig. 5B).

##### Description.

***Size and form***: Body length 1.7–2.1 mm. Body elongate oval, moderately convex (Fig. 5B). ***Color and punctation***: Dorsal surfaces of body dark brown, with clypeus (Fig. 2A), outer margins of pronotum, and outer margins of elytra yellowish brown (Fig. 5A, B); maxillary and labial palps yellow, remainder mouthparts orange; antennae yellowish brown; legs orange brown with paler (yellow) tarsi; ventral surfaces brown (Fig. 5C). Ground punctation on head, pronotum and elytra moderately marked (Figs 2A, 5A). ***Head***: Eyes in dorsal view with anterior margin oblique and anteriorly directed (Fig. 2A, B), and outer margins slightly bulging from outline of head; in lateral view, eyes not anteriorly emarginate (Fig. 2B). Maxillary palps as long as 0.8 × width of head. ***Thorax***: Elytra with all kinds of punctures similar in size and degree of impression, moderately aligned in rows, not forming grooves (Fig. 5A). Elevation of mesoventrite forming a very low transverse carina (Fig. 5C). Metaventrite with distinct median, longitudinal, glabrous area, 3 × longer than wide, extending along posterior half (Fig. 5C). ***Abdomen***: Abdominal ventrites uniformly and very densely pubescent (Fig. 5C). Aedeagus (Fig. 11D). Basal piece 2 × longer than a paramere; greatest width of a paramere nearly 0.6 × greatest width of median lobe; outer margins of parameres slightly sinuate, inner margins strongly sinuate; apex of paramere sharply acute, pointing towards longitudinal midline of aedeagus; median lobe roughly sagittate, medially with narrow emargination extending along apical fourth; gonopore situated nearly at apical third of median lobe.

##### Etymology.

Named with the Greek words *luteo*, meaning yellow, and *margo* meaning margin, in reference to the striking yellow band surrounding the marginal areas of these beetles.

##### Distribution.

This species is known from several localities along the northwestern edge of the Guiana Shield in Bolívar State, Venezuela. See Fig. 13.

##### Life history.

*Tobochares luteomargo* is found on rock seeps on granitic inselberg-like habitat. Some of these seeps may be very small, less than half a square meter in size. The largest series of this species were collected in seeps on which there was abundant apparent lichen growth, and specimens were often hiding under these growths (Fig. 15E).

#### Tobochares emarginatus

Taxon classificationAnimaliaColeopteraHydrophilidae

species group

B9756193-795A-551D-A3A8-B914A9482A0A

##### Diagnosis.

The *emarginatus* species group can be recognized by the oblique and posteriorly directed anterior margin of the eye in lateral view, which emarginates the eye to about a quarter of its dorsal width (Fig. 2C; Kohlenberg and Short 2017: fig. 6C–F), the presence of a broad, ovoid to diamond-shaped glabrous patch on the metaventrite (Fig. 5F), the dense and uniform distribution of the hydrofuge pubescence on the abdominal ventrites (Fig. 5F), and the moderate (in number and size) metatibial spines.

**Figure 6.**
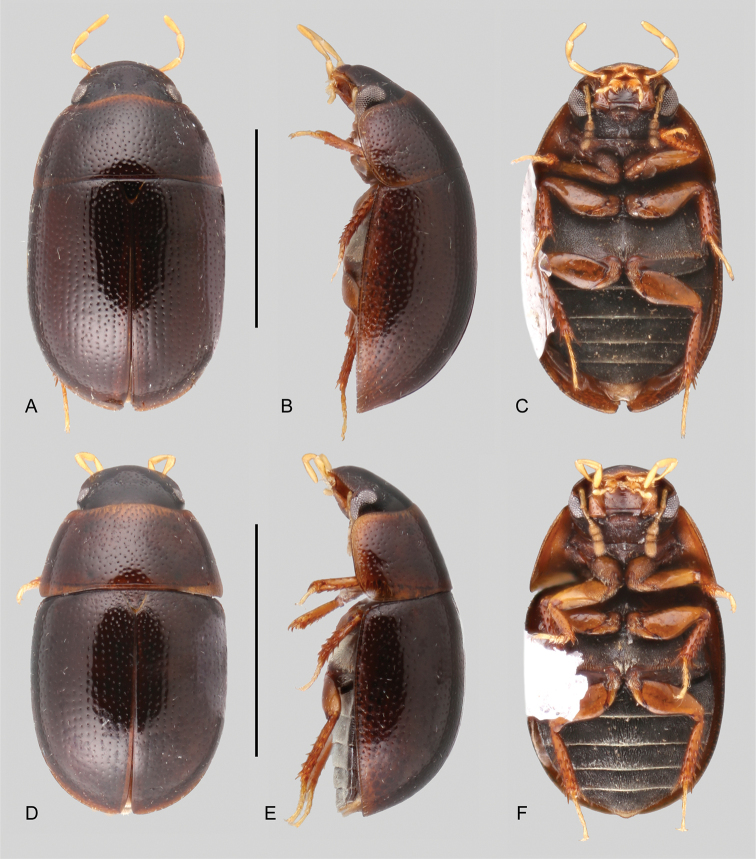
Habitus of *Tobochares* spp. in the *communis* species group **A–C***T. communis***A** dorsal view **B** lateral view **C** ventral view **D–F***T. microps***D** dorsal view **E** lateral view **F** ventral view. Scale bars: 1 mm.

##### Composition.

This species group presently contains three species: *T. canthus*, *T. emarginatus*, and *T. fusus* sp. nov.

**Figure 7.**
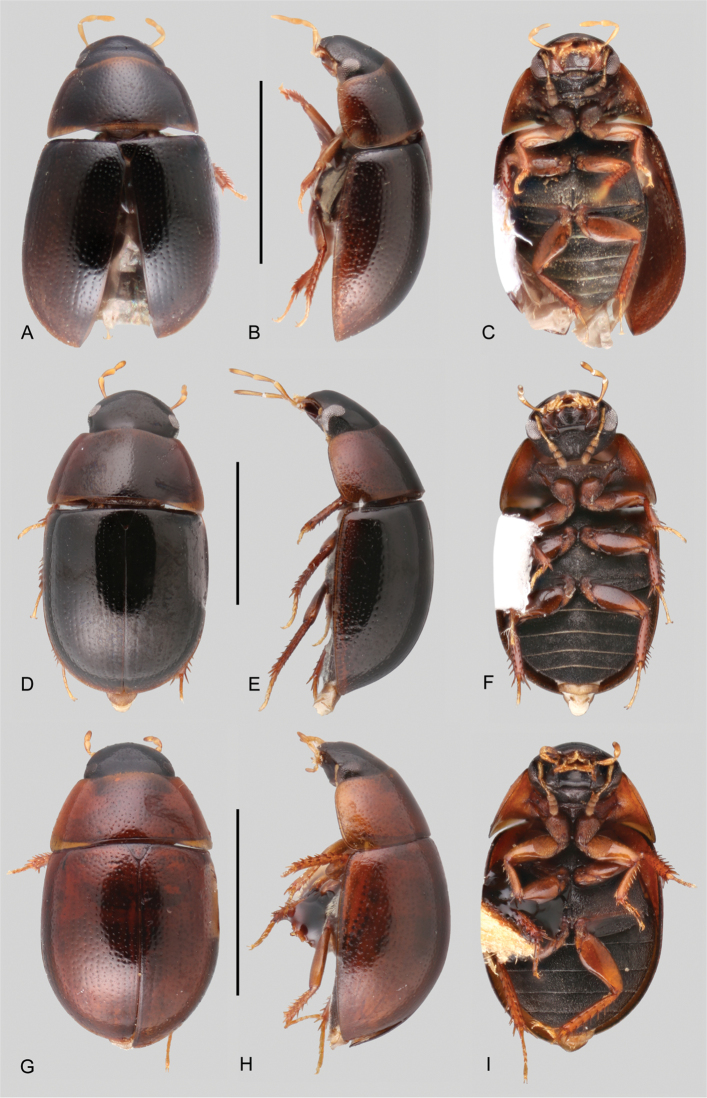
Habitus of *Tobochares* spp. in the *communis* species group **A–C***T. anthonyae***A** dorsal view **B** lateral view **C** ventral view **D–F***T. atures***D** dorsal view **E** lateral view **F** ventral view **G–I***T. pemon***G** dorsal view **H** lateral view **I** ventral view. Scale bars: 1 mm.

### Key to the species of *Tobochares emarginatus* species group

**Table:** 

1	Dorsal coloration light brown, rather uniform along body; serial rows of punctures on elytra very faintly defined (serial punctures slightly more impressed than ground punctures). Aedeagus with outer margin of parameres straight (Kohlenberg and Short 2017: fig. 14H) (Venezuela)	***Tobochares canthus* Kohlenberg & Short**
–	Dorsal coloration medium to dark brown, sometimes paler on pronotum; serial rows of punctures on elytra undefined (serial and ground punctures similarly impressed). Aedeagus with outer margin of parameres sinuate to convex (Brazil, Suriname, French Guiana)	**2**
2	Aedeagus slender, nearly 2.2 × longer than wide, with outer margin of parameres sinuate (parallel sided along basal two-thirds, then bending inward and tapering along apical third; Kohlenberg and Short 2017: fig. 14I)	***Tobochares emarginatus* Kohlenberg & Short**
–	Aedeagus fusiform, nearly 2 × longer than wide, with outer margin of parameres convex, (diverging along basal half, then broadly bending inward and tapering along apical fifth; see Fig. 11C)	***Tobochares fusus* sp. nov.**

#### Tobochares fusus sp. nov.

Taxon classificationAnimaliaColeopteraHydrophilidae

745AB8B1-3AFA-55B7-BAF8-0954868FD0F5

http://zoobank.org/6675298C-0C44-4D7A-9C50-29E620DEC24D

Figs 2C , 5D–F , 11C , 14F , 17

##### Type material examined.

***Holotype* (male)**: “Brazil: Amapá: Oiapoque/ 3.85039, -51.81683; 17 m/ Oiapoque (ca. 1 km E); 18.vii.2018/leg. Short; Flotation of detritus/ex forest seep; BR18-0718-03C” (INPA). ***Paratypes* (58 exs.): Brazil: Amapá**: Same data as holotype (45, INPA, MNHN, SCC, SEMC including DNA voucher SLE1564); Oiapoque (4 km NE), 3.87234, -51.80315, 14 m, 18.vii.2018, leg. Short, root mats on rock at margin seep, BR18-0718-02A (10, INPA, SEMC); Oiapoque (ca. 5.5 km NE), Balneario, 18.vii.2018, leg. Short, margin of larger stream, BR18-0718-01C (1, SEMC). **French Guiana**: Savane Roche Virginie, near RN 2, 4.1883, -52.13982, 64 m, 10.iii.2020, leg. Short and Neff, rotting *Clusia* fruits, FG20-0310-01D (3, SEMC including DNA Voucher SLE2171).

##### Differential diagnosis.

*Tobochares fusus* is externally indistinguishable from *T. emarginatus*, given that both species share the same disposition and degree of impression of the elytral punctures, and a low transverse ridge on the posterior elevation of the metaventrite. However, they can be clearly differentiated by the general shape of the aedeagus, which is rather slender and nearly parallel sided in *T. emarginatus* (Kohlenberg and Short 2017: fig. 14I), whereas *T. fusus* has a fusiform aedeagus (see Fig. 11C).

##### Description.

***Size and form***: Body length 1.7–2.0 mm. Body elongate oval, moderately convex (Fig. 5D, E). ***Color and punctation***: Dorsal surfaces of body dark brown, with slightly paler pronotum (Fig. 5D); antennae, mouthparts, and legs yellowish brown, with orange tibiae; ventral surfaces of meso-, metathorax and abdomen dark brown (Fig. 5F). Ground punctation on head, pronotum and elytra moderately marked. ***Head***: Eyes in dorsal view with anterior margin oblique, posteriorly directed; canthus emarginating eye to about a quarter of its dorsal width in lateral view (Fig. 2C). Maxillary palps 0.7 × width of head. ***Thorax***: Elytra with all kinds of punctures similar in size and degree of impression, seemingly evenly distributed, not forming grooves (Fig. 5D). Elevation of mesoventrite forming a low transverse ridge (Fig. 5F). Metaventrite with distinct median, broad, diamond-shaped glabrous area extending along posterior two thirds (Fig. 5F). ***Abdomen***: Abdominal ventrites uniformly and densely pubescent (Fig. 5F). Aedeagus (Fig. 11C). Basal piece nearly 0.4 × length of a paramere; greatest width of a paramere nearly 0.7 × greatest width of median lobe; outer margins of parameres diverging along basal half, then broadly bending inward and tapering along apical fifth; apex of paramere oblique, pointing towards longitudinal midline of aedeagus; median lobe roughly triangular, apically rounded; gonopore situated at apex of median lobe.

##### Etymology.

Named with the Latin word *fusus*, meaning fusiform, in reference to the shape of the aedeagus of this species.

##### Distribution.

This species is known from two closely situated localities on either side of the Oiapoque River, the boundary between French Guiana and the Brazilian state of Amapá. See Fig. 13.

##### Life history.

The series from Brazil were taken from a seepage habitat in a forested riparian corridor (Fig. 14F). A thin layer of saturated dead leaves was laying over granite, over which a thin film of water was seeping into an adjacent stream. The short series from French Guiana was collected from the rotting fruits of a *Clusia* on an otherwise dry forest floor and not adjacent to any aquatic habitat (Fig. 17C).

#### Tobochares communis

Taxon classificationAnimaliaColeopteraHydrophilidae

species group

E1FB953C-41AE-5865-B61B-61D56B7AFAD8

##### Diagnosis.

The *Tobochares communis* species group can be recognized by the straight anterior margin of the eye in lateral view (e.g., Fig. 2E), the usually longitudinally aligned elytral punctures, the short and narrow glabrous patch on the metaventrite (Fig. 3D, E), the high density of the hydrofuge pubescence on the abdominal ventrites, and the well-developed and numerous metatibial spines.

##### Composition.

*Tobochares akoerio* sp. nov., *T. arawak* sp. nov., *T. anthonyae* sp. nov., *T. atures* sp. nov., *T. canaima* sp. nov., *T. communis* sp. nov., *T. kappel* sp. nov., *T. kolokoe* sp. nov., *T. microps* sp. nov., *T. pemon* sp. nov., and *T. romanoae* sp. nov.

### Key to the species of *Tobochares communis* species group

**Table:** 

1	Elytra with all kinds of punctures relatively large, about the same size and degree of impression, all seemingly longitudinally aligned and uniformly distributed (Fig. 6A, D)	**2**
–	Elytra with serial punctures either larger, denser or more impressed (or a combination of those features) than the remainder punctures; interserial punctures either similar in size, smaller, denser, longitudinally aligned or irregularly distributed regarding serial punctures (e.g. Figs 7A, D, G, 8A, D, 9A, D, 10A, D)	**3**
2	Eyes relatively small (ventral face of the eye only slightly wider than antennal club), separated by distance 6 × larger than largest diameter of eye in dorsal view (Fig. 2I)	***Tobochares microps***
–	Eyes of normal size (ventral face of the eye nearly twice as wide as antennal club), separated by distance 4.5 × larger than largest diameter of eye in dorsal view (Fig. 2H)	***Tobochares communis***
3	General coloration orange brown with black head (Fig. 7G, H); all elytral punctures similar in size and degree of impression, most equidistant from each other; serial punctures only seemingly longitudinally aligned (Figs 3F, 7G); aedeagus with apex of median lobe emarginate (Fig. 11E)	***Tobochares pemon***
–	General coloration dark brown; elytral punctation variable; aedeagus with apex of median lobe variable, but never emarginate	**4**
4	Surface of elytra sharply impressed along rows of serial punctures, forming well-defined striae along posterior 2/3 of elytra (Fig. 8A, D)	**5**
–	Surface of elytra not or only slightly and uniformly impressed, forming shallow grooves along entire rows of serial punctures (e.g., Fig. 7A)	**6**
5	Body size around 2.6 mm; elytral striae rather shallow (Fig. 8A, B); mesofemur with a well-defined patch of dense hydrofuge pubescence along anterobasal corner (Fig. 8C); metafemur 2.5 × longer than wide (Fig. 8C)	***Tobochares romanoae***
–	Body size around 2.0 mm; elytral striae rather broad and deep (Fig. 8D, E); mesofemur with a reduced patch of scanty hydrofuge pubescence on anterobasal corner (Fig. 8F); metafemur nearly 2 × longer than wide (Fig. 8F)	***Tobochares akoerio***
6	Serial punctures longitudinally aligned and slightly impressed forming shallow grooves (Fig. 7A); interserial punctures somewhat irregularly distributed in a single row (Fig. 7A)	***Tobochares anthonyae***
–	Serial punctures never impressed to form grooves; interserial punctures variable in distribution	**7**
7	Serial punctures longitudinally aligned, larger than interserial punctures (Fig. 3B, G); interserial punctures longitudinally aligned, more densely arranged than serial punctures (Fig. 3B, G); median lobe gradually narrowing towards apex from near base (Fig. 11G)	***Tobochares atures***
–	Serial punctures seemingly longitudinally aligned, more densely arranged than interserial punctures (e.g., Fig. 3C); interserial punctures randomly distributed (e.g., Fig. 3H, I); median lobe broad, only narrowing along apical third (e.g., Fig. 11L–N)	**8**
8	Elytra with 1 or 2 irregular rows of interserial punctures (elytral punctation moderately dense, Figs 3H, 9A, D); tibiae orange to orange brown in coloration; maxillary palps bright yellow	**9**
–	Elytra with 2 or 3 irregular rows of interserial punctures (elytral punctation highly dense, Figs 3I, 10A, D); tibiae reddish brown in coloration; maxillary palps tan yellow	**10**
9	Lateral coloration of pronotum and elytra gradually paler, orange (Fig. 9B); legs orange (Fig. 9C); serial punctures along outer surface of elytra equally impressed as those on dorsal surface (Fig. 9B)	***Tobochares kappel***
–	Coloration of pronotum only slightly paler along antero-lateral margin (Fig. 9E); legs reddish brown (Fig. 9F); punctures on outer surface of elytra more strongly impressed along apical region than those on dorsal surface (Fig. 9F)	***Tobochares kolokoe***
10	Elytral punctation sharp and dense (Fig. 10D); apodemes of the median lobe one fourth the length of the median lobe (Fig. 11N)	***Tobochares canaima***
–	Elytral punctation shallow and relatively sparse (Fig. 10A); apodemes of the median lobe nearly half as long as the median lobe (Fig. 11M)	***Tobochares arawak***

#### Tobochares akoerio sp. nov.

Taxon classificationAnimaliaColeopteraHydrophilidae

8FC0F660-8760-531E-944D-48839959073A

http://zoobank.org/E8A50621-1E33-48BC-8331-1C2C5F071BAA

**Figure 8.**
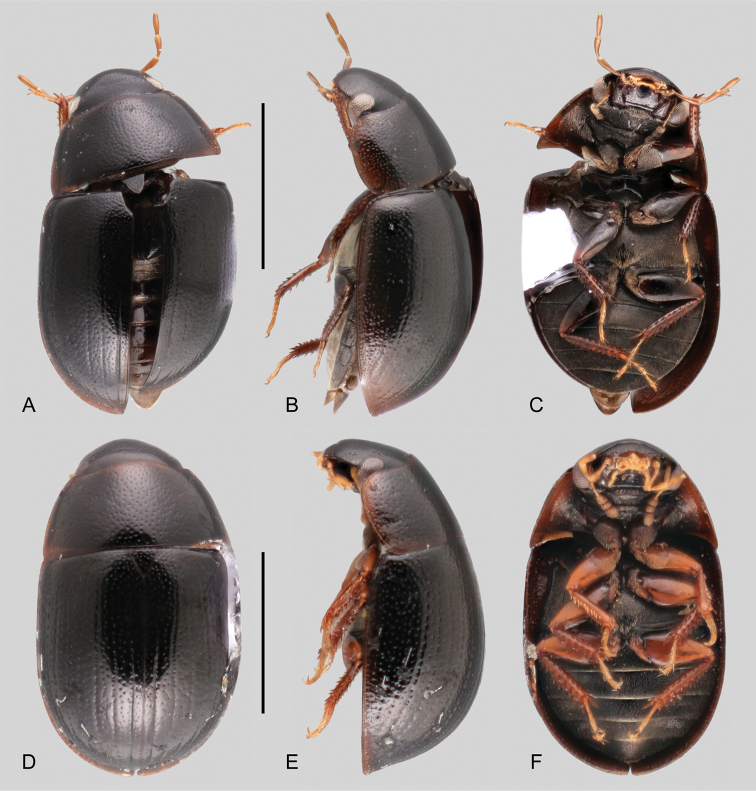
Habitus of *Tobochares* spp. in the *communis* species group **A–C***T. romanoae***A** dorsal view **B** lateral view **C** ventral view **D–F***T. akoerio***D** dorsal view **E** lateral view **F** ventral view. Scale bars: 1 mm.

Figs 8D–F , 11K , 13

##### Type material examined.

***Holotype* (male)**: “Suriname: Sipaliwini District, 2.46554°N, 55.7700°; 800 m; Camp 2, Grensgebergte Rock; rock seepages; 12.iii.2012; leg. A. Short; SR12-0312-01A” (NZCS). ***Paratypes* (5 exs.)**: Same data as holotype (5, SEMC).

##### Differential diagnosis.

*Tobochares akoerio* can be recognized by its strongly convex body in lateral view (Fig. 8E), accompanied by elytra with well-defined rows of serial punctures, moderately impressed, forming grooves along apical 3/4 of elytra (Fig. 8D, E); interserial punctures somewhat irregularly distributed (Fig. 8D, E). The general habitus of *T. akoerio* is similar to that of *T. romanoae* and *T. canaima*, especially by the uniformly dark coloration of the pronotum; *T. akoerio* can be distinguished from these two species by its strongly impressed striae, especially along the lateral regions of the elytra when compared to *T. romanoae* (compare Fig. 8D, E to 8A, B).

**Figure 9.**
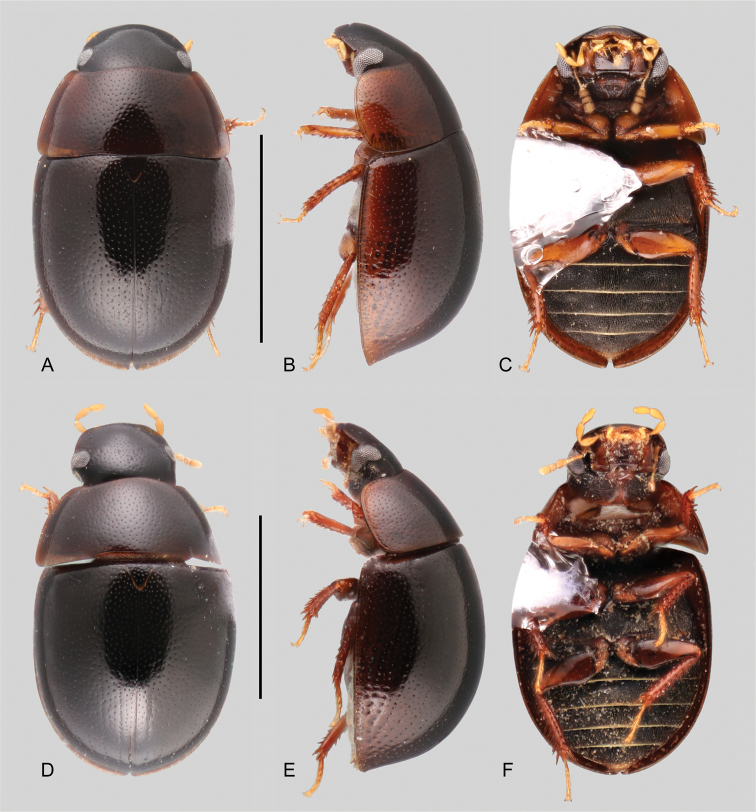
Habitus of *Tobochares* spp. in the *communis* species group **A–C***T. kappel***A** dorsal view **B** lateral view **C** ventral view **D–F***T. kolokoe***D** dorsal view **E** lateral view **F** ventral view. Scale bars: 1 mm.

##### Description.

***Size and form***: Body length 2.0 mm. Body elongate oval, strongly convex (Fig. 8E). ***Color and punctation***: Dorsal and ventral surfaces of body dark brown, with lateral margins of prothorax only very slightly paler (Fig. 8D, E); mouthparts and antennae yellow, with slightly darker antennal club and apical third of maxillary palpomere IV; legs orange with paler tarsi (Fig. 8F). Ground punctation on head, pronotum and elytra moderately marked (Fig. 8D). ***Head***: Eyes in dorsal view with anterior margin oblique (anteriorly directed), and outer margins slightly bulging from outline of head; in lateral view, eyes not emarginate. ***Thorax***: Elytra with well-defined rows of serial punctures, moderately impressed, forming grooves along apical 3/4 of elytra (Fig. 8D); interserial punctures somewhat irregularly distributed (Fig. 8D). Metafemora mostly glabrous on anterior face, with narrow band of pubescence along basal third of dorsal margin (Fig. 8D). Elevation of mesoventrite forming a low transverse carina (Fig. 8D). Metaventrite with distinct median, longitudinal, narrow glabrous area extending along posterior half (Fig. 8D). ***Abdomen***: Abdominal ventrites uniformly and very densely pubescent. Aedeagus (Fig. 11K). Basal piece 0.4 × the length of a paramere; parameres nearly 1/3 as narrow as greatest width of median lobe, with outer margins widely and uniformly convex, and rounded apex; median lobe roughly triangular, rounded and slightly pinched at apex; gonopore situated nearly at midlength of median lobe.

##### Etymology.

Noun in apposition. Named after the Akoerio, an indigenous nomadic tribe, with only few people remaining in the South of Suriname.

##### Distribution.

The species is only known from an exposed rocky summit in the Grensgebergte Mountains along the border between Suriname and Brazil. See Fig. 13.

##### Life history.

This species was collected on flowing seeps with moss and algae over granite. See Fig. 16F.

#### Tobochares arawak sp. nov.

Taxon classificationAnimaliaColeopteraHydrophilidae

373AD260-35E5-5DE8-8C1F-496B981C165A

http://zoobank.org/11FEE7B8-26B6-4CD5-8447-2470751E1A4F

**Figure 10.**
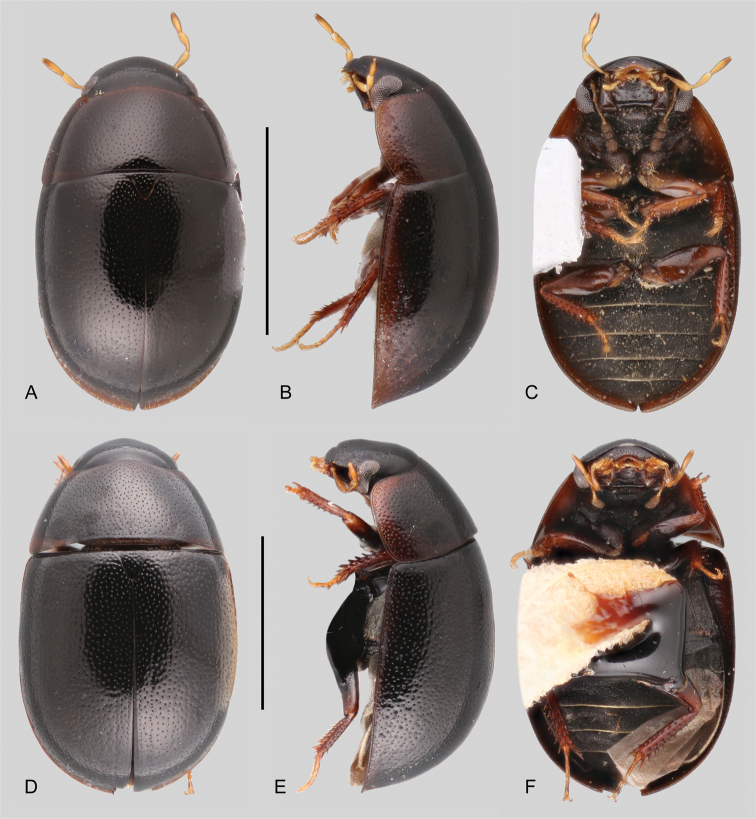
Habitus of *Tobochares* spp. in the *communis* species group **A–C***T. arawak***A** dorsal view **B** lateral view **C** ventral view **D–F***T. canaima***D** dorsal view **E** lateral view **F** ventral view. Scale bars: 1 mm.

**Figure 11.**
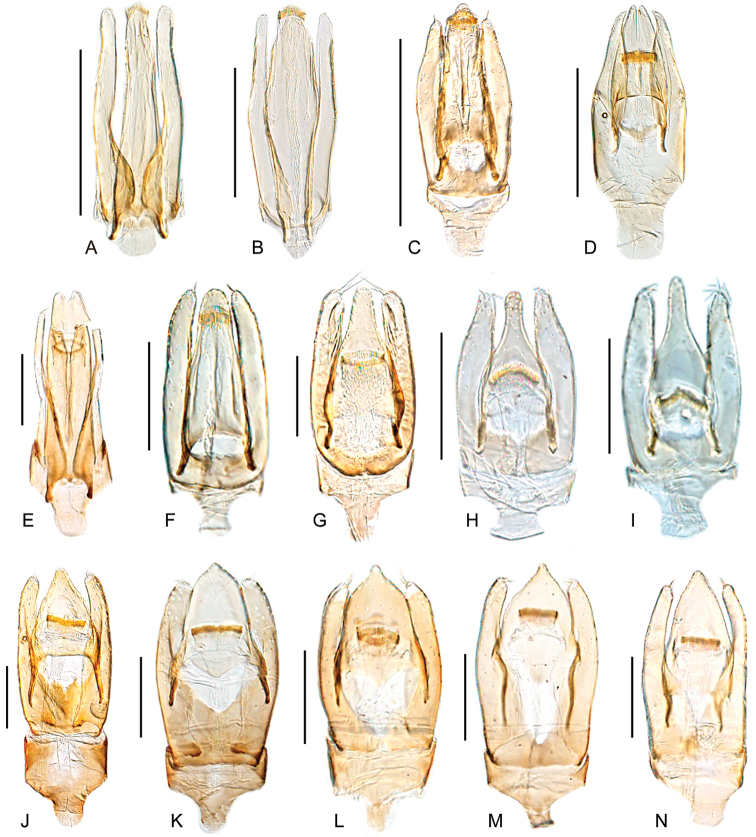
Aedeagi of *Tobochares* spp. **A***T. benettii***B***T. goias***C***T. fusus***D***T. luteomargo***E***T. pemon***F***T. anthonyae***G***T. atures***H***T. communis***I***T. microps***J***T. romanoae***K***T. akoerio***L***T. kappel***M***T. arawak***N***T. canaima*. Scale bars: 0.5 mm (**A–D**); 0.1 mm (**E–N**).

Figs 3C, E , 10A–C , 11M , 13 , 15A, B

##### Type material examined.

***Holotype* (male)**: “Guyana: Region VIII: 5°0.730'N, 59°38.965'W; 585 m; Upper Potaro Camp I; ca. 7 Km NW of Chenapau; top of falls on Potaro River; seeps with roots and algae; 12.iii.2014; leg. Short, Salisbury, La Cruz; GY14-0312-01B” (CBDG). ***Paratypes* (127 exs.): Guyana: Region VIII**: Same data as holotype (127, CBDG, SEMC).

##### Differential diagnosis.

*Tobochares arawak* can be recognized by its strongly convex body in lateral view (Fig. 10B), accompanied by elytral punctation uniform in size and degree of impression, with serial punctures seemingly aligned in rows, not impressed to form grooves (Fig. 10A); the interserial punctures are somewhat irregularly distributed in two or three rows (Fig. 10A). The general habitus and punctation of *T. arawak* are similar to those of *T. canaima*, *T. kappel*, and *T. kolokoe*. In *T. kappel* and *T. kolokoe* the interserial punctures form only one or two irregular rows (Fig. 3H). In *T. canaima* (Fig. 10D) the pronotal and elytral punctations are sharper than in *T. arawak* and the apodemes of the median lobe are one fourth the length of the median lobe in *T. canaima* (Fig. 11N), as opposed to half as long in *T. arawak* (Fig. 11M).

##### Description.

***Size and form***: Body length 1.6–1.8 mm. Body elongate oval, strongly convex (Fig. 10A, B). ***Color and punctation***: Dorsal and ventral surfaces of body dark brown, with lateral margins of prothorax and elytra only slightly paler (Fig. 10A, B); mouthparts yellow, with slightly darker apical third of maxillary palpomere IV; antennae brown; legs reddish to dark brown with paler tarsi (Fig. 10C). Ground punctation on head, pronotum and elytra moderately marked (Fig. 10A, B). ***Head***: Eyes in dorsal view with anterior margin oblique (anteriorly directed), and outer margins slightly bulging from outline of head; in lateral view, eyes not emarginate (see Fig. 2E). ***Thorax***: Elytra with slightly defined rows of shallow serial punctures, not forming grooves (Fig. 10A); interserial punctures somewhat irregularly distributed in two or three rows (Fig. 3I). Elevation of mesoventrite forming a very low transverse carina (Fig. 10C). Metaventrite with distinct median, longitudinal, narrow glabrous area extending along posterior half (Fig. 10C). ***Abdomen***: Abdominal ventrites uniformly and densely pubescent. Aedeagus (Fig. 11M). Basal piece 0.4 × the length of a paramere; parameres nearly 1/3 as narrow as greatest width of median lobe, with outer margins widely and uniformly convex, and rounded apex; median lobe roughly triangular, rounded and slightly pinched at apex; gonopore situated nearly at midlength of median lobe.

##### Etymology.

Noun in apposition. Named after the Arawak, an indigenous tribe of northern South America.

##### Distribution.

*Tobochares arawak* is only known from the Upper Potaro region in Guyana. See Fig. 13.

##### Life history.

This species was collected in a wet seepage area along rocks at the margin of the Upper Potaro River. Specimens were collected by pulling back root mats and moss that were growing over the wet rock areas. See Fig. 15A, B.

**Figure 12.**
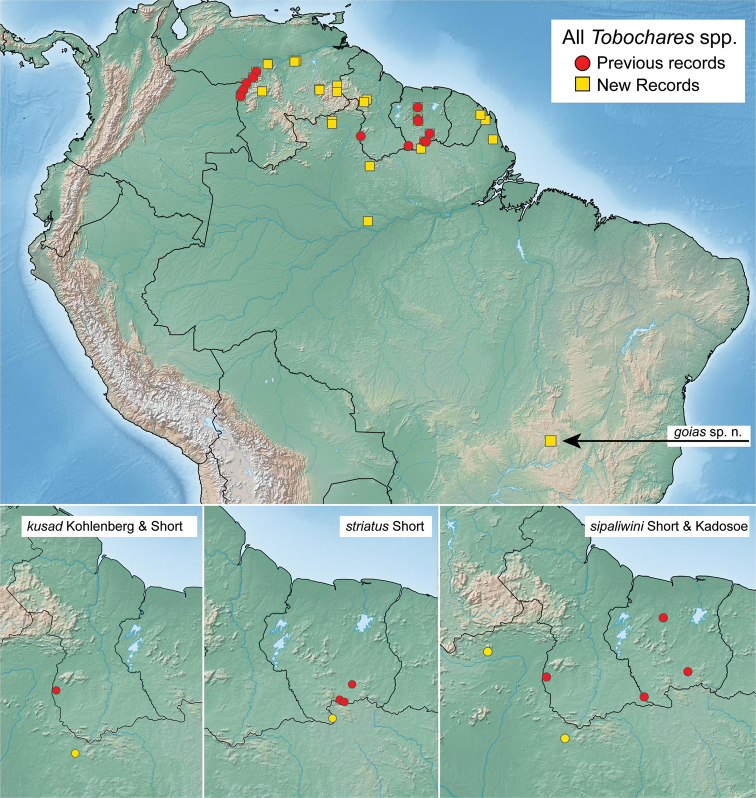
Distribution of *Tobochares* spp., including all previous (red) and new (yellow) records.

**Figure 13.**
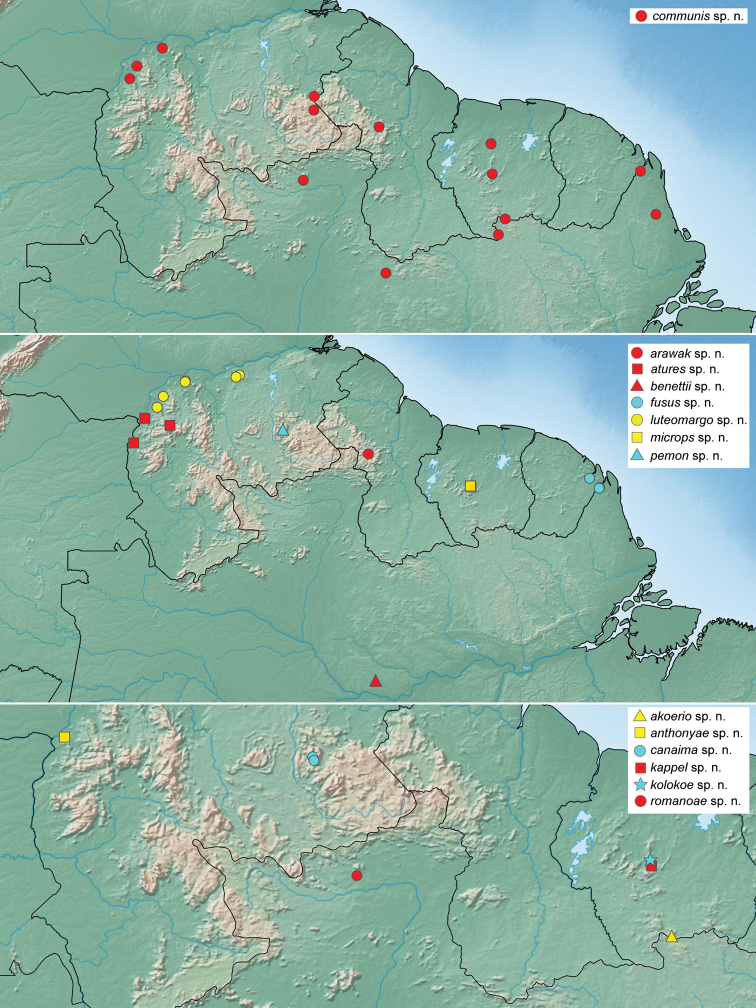
Distribution of *Tobochares* spp.

**Figure 14.**
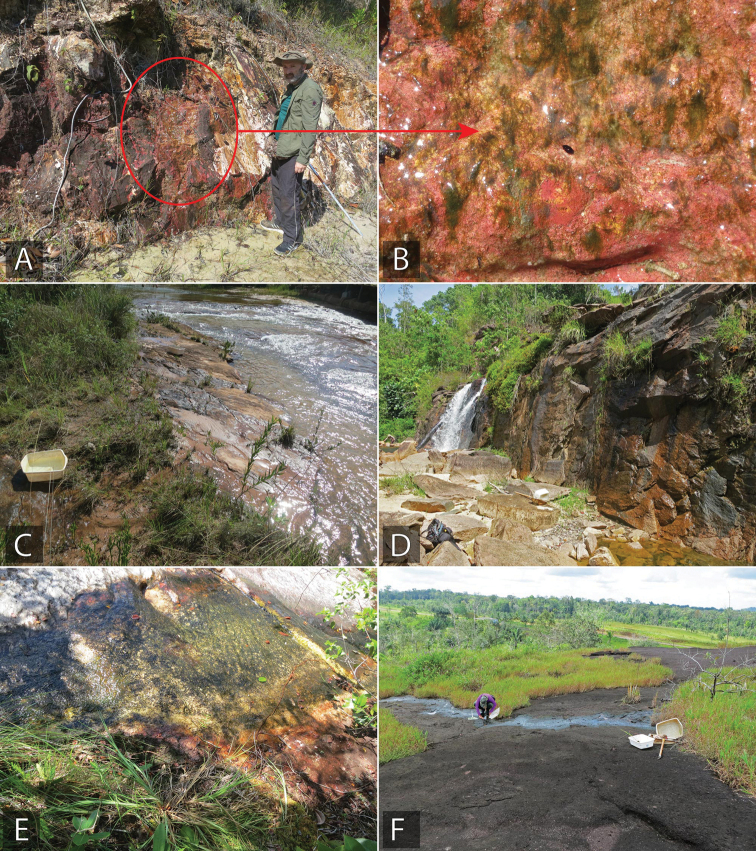
*Tobochares* habitat in Brazil **A, B** type locality and habitat for *T. benettii*, seepage near Rio Preto de Eva (collecting event BR17-0610-01A) **C** type locality and habitat for *T. goias*, margin of Balneario Lejas (collecting event BR18-0304-02B) **D** habitat of *T. kusad* and *T. sipaliwini*, State of Roraima, near Usina de Jatapú reservoir (collecting Event BR18-0117-01A) **E** habitat and type locality of *T. romanoae*, and habitat of *T. sipaliwini*, State of Roraima, Serra do Tepequém, Igarape Preto Negro, Cachoeira Leje Preta (collecting event BR18-0114-04B) **F** type locality and habitat of *T. fusus*, State of Amapá, Calcoene (collecting event BR18-0721-02B).

#### Tobochares anthonyae sp. nov.

Taxon classificationAnimaliaColeopteraHydrophilidae

5429D85B-D30B-57C3-BB2A-E8D7FC4BE1AF

http://zoobank.org/DBADDDCB-34F7-4608-810E-D488FBBD3ABE

Figs 7A–C , 11F , 13

##### Type material examined.

***Holotype* (male)**: “Venezuela: Bolívar: 6°13'4.6"N, 67°14'26.4"W; 60 m; ca. 25 Km E of El Burro; rocky morichal; 12.i.2009; leg. Short et al.; VZ09-0113-01X” (MIZA). ***Paratypes* (3 exs.): Venezuela: Bolívar**: “6°13'4.6"N, 67°14'26.4"W; 60 m; ca. 25 Km E of El Burro; rocky morichal; 7.viii.2008; leg. Short, García, Joly; AS-08-077” (1, SEMC); same data as holotype (2, SEMC).

##### Differential diagnosis.

The general habitus and coloration of *T. anthonyae* is similar to that of several species in the *communis* group; nevertheless, the elytral punctation *T. anthonyae* is relatively distinct: all kinds of punctures are relatively large, similar in size and degree of impression, the serial punctures are aligned in rows and slightly impressed, forming shallow longitudinal grooves, and the interserial punctures are somewhat irregularly distributed in a single row (Fig. 7A). The relatively large punctures, similar in size and degree of impression may resemble those of *T. communis*, but in this species the serial punctures are not impressed to form grooves (Fig. 6A), as they are in *T. anthonyae*. In addition, the overall shape of the aedeagus, especially the shape of the median lobe of *T. anthonyae* is unique among members of the *communis* group: the median lobe gradually and slightly narrows towards a broadly rounded apex, and the gonopore is located near the apex of the median lobe (Fig. 11F).

##### Description.

***Size and form***: Body length 1.8–2.0 mm. Body elongate oval, moderately convex (Fig. 7B). ***Color and punctation***: Dorsal and ventral surfaces of body, dark brown, with slightly paler margins of pronotum (Fig. 7A, B); mouthparts yellowish brown; antennae light brown; legs orange with yellow tarsi (Fig. 7C). Ground punctation on head, pronotum and elytra rather shallowly marked. ***Head***: Eyes in dorsal view with anterior margin oblique (anteriorly directed; e.g., Fig. 2D), and outer margins slightly bulging from outline of head; in lateral view, eyes not anteriorly emarginate (e.g., Fig. 2E). ***Thorax***: Elytra with all kinds of punctures similar in size and degree of impression (Fig. 7A); serial punctures aligned in rows, slightly impressed, forming shallow longitudinal grooves; interserial punctures somewhat irregularly distributed in a single row (Fig. 7A). Metafemora mostly glabrous on anterior face, with hydrofuge pubescence along basal third of antero-dorsal margin (Fig. 7C). Elevation of mesoventrite forming a low longitudinal bulge (Fig. 7C). Metaventrite with distinct median, longitudinal, narrow glabrous area extending along posterior half (Fig. 7C). ***Abdomen***: Abdominal ventrites uniformly and very densely pubescent. Aedeagus (Fig. 11F). Basal piece 0.3 × the length of a paramere; greatest width of a paramere nearly 0.5 × greatest width of median lobe; outer margins of parameres nearly straight, only slightly curved inwards along apical region; apex of paramere rounded; median lobe roughly triangular, widely rounded at apex; gonopore situated at apical fourth of median lobe.

##### Etymology.

Named after Becky Anthony, program and meetings manager at the Entomological Society of America (ESA), in recognition of all her hard work in service to the society and the entomological community.

##### Distribution.

Only known from a single locality just south of the Orinoco River along the northwestern edge of the Guiana Shield. See Fig. 13.

##### Life history.

The specimens were collected along a stream that was flowing over exposed granite.

#### Tobochares atures sp. nov.

Taxon classificationAnimaliaColeopteraHydrophilidae

21DDD52D-BD4F-5409-AE90-1B50F0236358

http://zoobank.org/EA1F3EA4-DD9D-4A07-8CB1-219E91CAD0AA

**Figure 15.**
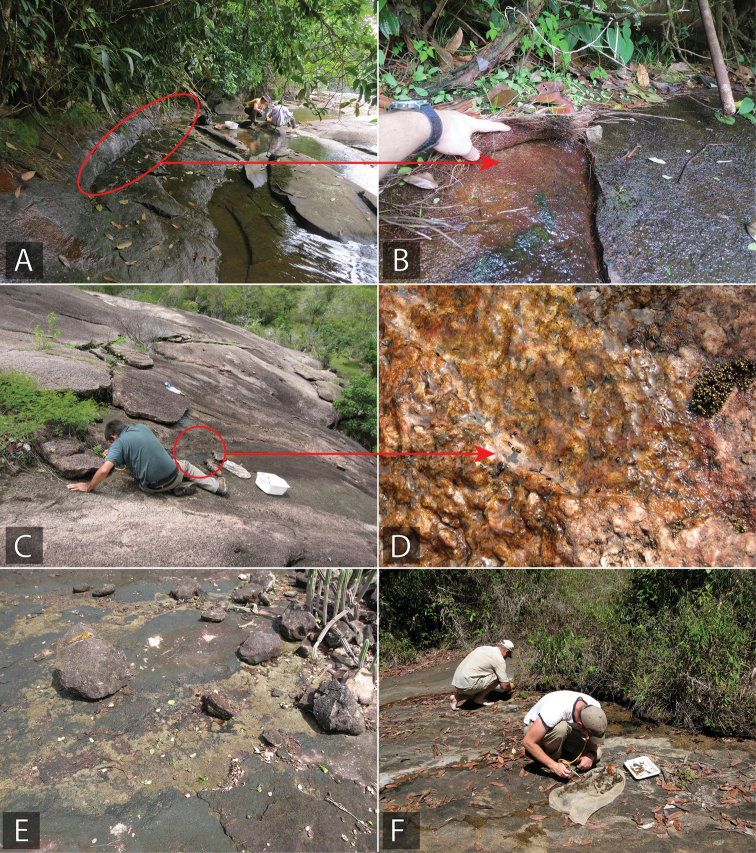
*Tobochares* habitat in Guyana and Venezuela **A, B** habitat for *T. arawak*, Guyana, upper Potaro River (collecting event GY14-0312-01B) **C, D** habitat for *T. luteomargo*, Venezuela, rock outcrop by Río Cuchivero, (collecting event VZ10-0710-01A) **E** habitat for *T. luteomargo*, Venezuela Campamento Río Aro (collecting event VZ10-0711-01B) **F** type locality and habitat for *T. atures*, Venezuela, Tobogan de la Selva (collecting event VZ09-0114-01F).

Figs 3B, G , 7D, F , 11G , 13 , 15F

Tobochares sp. 8: Short et al. (2021)

##### Type material examined.

***Holotype* (male): Venezuela**, “T.F. Amazonas/ Puerto Ayacucho (40km S)/ El Tobogán, Caño Coromoto/ 26 Jan 1989, stream edge/ at upper shelter”, “collected by/ PJSpangler/RAFaitoute & CBBarr” (MIZA). ***Paratypes* (357 exs.): Venezuela: Amazonas**: “40 Km S of Puerto Ayacucho, at Tobogán; upper seep; 18.i.1989; leg. Spangler, Faitoute, Barr” (34, USNM); same, except “; colln #1; collected by pouring water over stream bank and washing riparian insects into seine; 19.vi.1989; leg. Spangler and Faitoute” (3, USNM); same, except “sandy margins; colln. #10; 23.ii.1986; leg. P. Spangler” (38, USNM); same, except “colln. #14; 25.ii.1986” (16, USNM); “40 Km S of Puerto Ayacucho, El Tobogán, Caño Coromoto; seep, at upper shelter; 26.i.1989; leg. Spangler, Faitoute, Barr” (55, USNM); same data as holotype (174, SEMC, MIZA, USNM); “5°62'N [Sic!], 66°23'W; 1250 m; Cerro Guanay; Exp. Terramar; 5–12.ii.1995; leg. J. Clavijo” (1, MIZA); Tobogán de la Selva; old “Tobogancito” on seepage area with detritus; 8.viii.2008; leg. Short, García, Joly; AS-08-080b (16, SEMC including DNA voucher SLE1032); same, except “Tobogán de la Selva; wet rock covered with detritus; upstream slide; 14.i.2009; leg. Short, García, Miller, Joly; VZ09-0114-01F” (16, SEMC); same, except “partly shaded wet rock with algae; leg. Short and Miller; VZ09-0114-01G” (2, SEMC). **Bolívar**: “6°13'4.6"N, 67°14'26.4"W; 60 m; ca. 25 Km E of El Burro; rocky morichal; 12.i.2009; leg. Short and Téllez; VZ09-0113-01X” (1, SEMC).

##### Differential diagnosis.

The general habitus and coloration of *T. atures* is similar to that of several species in the *communis* group, nevertheless, the elytral punctation in *T. atures* is relatively distinct: all the elytral punctures are shallowly impressed, longitudinally aligned, and have two different sizes: the serial punctures, which are slightly impressed, are larger, whereas the interserial punctures are smaller and denser (Fig. 3B, G). In addition, the overall shape of the aedeagus, especially the shape of the median lobe of *T. atures* is unique among members of the *communis* group: the median lobe gradually and slightly narrows towards a rounded apex, and the gonopore is located at the apical third of the median lobe (Fig. 11G).

##### Description.

***Size and form***: Body length 2.0–2.2 mm. Body elongate oval, moderately convex (Fig. 7D). ***Color and punctation***: Dorsal and ventral surfaces of body dark brown, with prothorax and lateral margins of elytra slightly paler (Fig. 7D, E); mouthparts and antennae yellow, with slightly darker apical third of maxillary palpomere IV; legs orange to reddish brown with paler tarsi (Fig. 7E). Ground punctation on head, pronotum and elytra rather shallowly marked. ***Head***: Eyes in dorsal view with anterior margin oblique (anteriorly directed), and outer margins slightly bulging from outline of head (e.g., Fig. 2D); in lateral view, eyes not emarginate (Fig. 2E). ***Thorax***: Elytra with longitudinal rows of shallow punctures, not forming grooves; punctures in two different sizes: serial punctures larger, interserial punctures smaller and denser (Fig. 3B, G). Metafemora mostly glabrous on anterior face (Fig. 7F). Elevation of mesoventrite forming a low transverse carina (Fig. 7F). Metaventrite with distinct median, longitudinal, narrow glabrous area extending along posterior half (Fig. 7F). ***Abdomen***: Abdominal ventrites uniformly and very densely pubescent (Fig. 7F). Aedeagus (Fig. 11G). Basal piece nearly 0.5 × the length of a paramere; parameres nearly 0.4 × greatest width of median lobe, with outer margins weakly and uniformly convex, and rounded apex; median lobe roughly triangular, widely rounded at apex; gonopore situated nearly at apical third of median lobe.

##### Etymology.

Noun in apposition. Named after Atures, the municipality where the type locality is situated.

##### Distribution.

This species is known from several localities along the northwestern edge of the Guiana Shield in Venezuela. See Fig. 13.

##### Life history.

Most specimens were collected on granite seepages that were adjacent to permanent streams. See Fig. 15F.

#### Tobochares canaima sp. nov.

Taxon classificationAnimaliaColeopteraHydrophilidae

4EF0B019-48C7-579E-8E3F-C4EE79583DCF

http://zoobank.org/D344B5AF-D885-4015-BAE5-0F7AF163E24A

Figs 3I , 10D–F , 11N , 13

##### Type material examined.

***Holotype* (male)**: “Venezuela: Bolívar: 5°51'N, 62°33'W; 1700 m; Auyan-tepui; Intercept trap; 7–14.ii.1994; leg. J.L. García, A. Chacón” (MIZA). ***Paratypes* (7 exs.): Venezuela: Bolívar**: Same data as holotype (6, MIZA, SEMC); “5°46'50"N, 62°31'36"W; 2170 m; Auyan-tepui; yellow trap; 20.iv.1994; leg. L. Mesner, J.L. García” (1, MIZA).

##### Differential diagnosis.

*Tobochares canaima* can be recognized by its strongly convex body in lateral view, accompanied by elytral punctation uniform in size and degree of impression, with serial punctures seemingly aligned in rows, not impressed to form grooves; the interserial punctures are somewhat irregularly distributed in two or three rows (Fig. 10D, E). The general habitus and punctation of *T. canaima* are similar to those of *T. arawak*, *T. kappel*, and *T. kolokoe*. In *T. kappel* and *T. kolokoe* the interserial punctures form only one or two irregular rows (e.g., Fig. 3H). In *T. arawak* the pronotal and elytral punctation is shallower than in *T. canaima* (compare Fig. 10A vs. Fig. 10B) and the apodemes of the median lobe are half the length of the median lobe in *T. arawak* (Fig. 11M), as opposed to one fourth of the length in *T. canaima* (Fig. 11N).

##### Description.

***Size and form***: Body length 1.9–2.1 mm. Body elongate oval, moderately convex (Fig. 10E). ***Color and punctation***: Dorsal and ventral surfaces of body dark brown, with anterolateral margins of prothorax slightly paler (Fig. 10D, E); mouthparts yellow to orange; antennae light brown; legs reddish to dark brown, distally paler (orange), with paler (yellow) tarsi (Fig. 10F). Ground punctation on head, pronotum and elytra sharply marked; pronotal punctation dense (Fig. 10D). ***Head***: Eyes in dorsal view with anterior margin oblique (anteriorly directed; e.g., Fig. 2D), and outer margins slightly bulging from outline of head; in lateral view, eyes not emarginate (e.g., Fig. 2E). ***Thorax***: Elytra with serial punctures similar in size and degree of impression to interserial punctures, and only seemingly aligned longitudinally, not forming grooves; interserial punctures irregularly distributed in two or three rows (Fig. 10D). Metafemora mostly glabrous on anterior face (Fig. 10F). Elevation of mesoventrite forming a very low transverse carina (Fig. 10F). Metaventrite with distinct median, longitudinal, narrow glabrous area extending along posterior half (Fig. 10F). ***Abdomen*.** Abdominal ventrites uniformly and very densely pubescent (Fig. 10F). Aedeagus (Fig. 11N). Basal piece nearly 0.45 × the length of a paramere; greatest width of a paramere nearly 0.6 × greatest width of median lobe; outer margins of parameres straight to widely and uniformly convex; apex of paramere obliquely rounded; median lobe roughly sagittate, rounded at apex; gonopore situated nearly at midlength of median lobe.

##### Etymology.

Noun in apposition. Named after the Canaima National Park in Venezuela, where the type locality is situated.

##### Distribution.

This species is known from the famous Auyan-tepui, which is also home to Angel Falls, the highest waterfall in the world. Collected at elevations of 1700–2170 m, this species is one of the relatively few water beetle taxa known from the “Pantepui Province”, which comprises areas of the Guiana Shield which are greater than 1500 m in elevation. See Fig. 13.

##### Life history.

The only known series was collected in a flight intercept trap and a yellow pan trap. Nothing further is known about the habitat or biology of this species.

#### Tobochares communis sp. nov.

Taxon classificationAnimaliaColeopteraHydrophilidae

533C525A-E3A4-500A-B7AA-779059209867

http://zoobank.org/FBCAB8BF-C5FE-4C4B-9681-17621C615829

**Figure 16.**
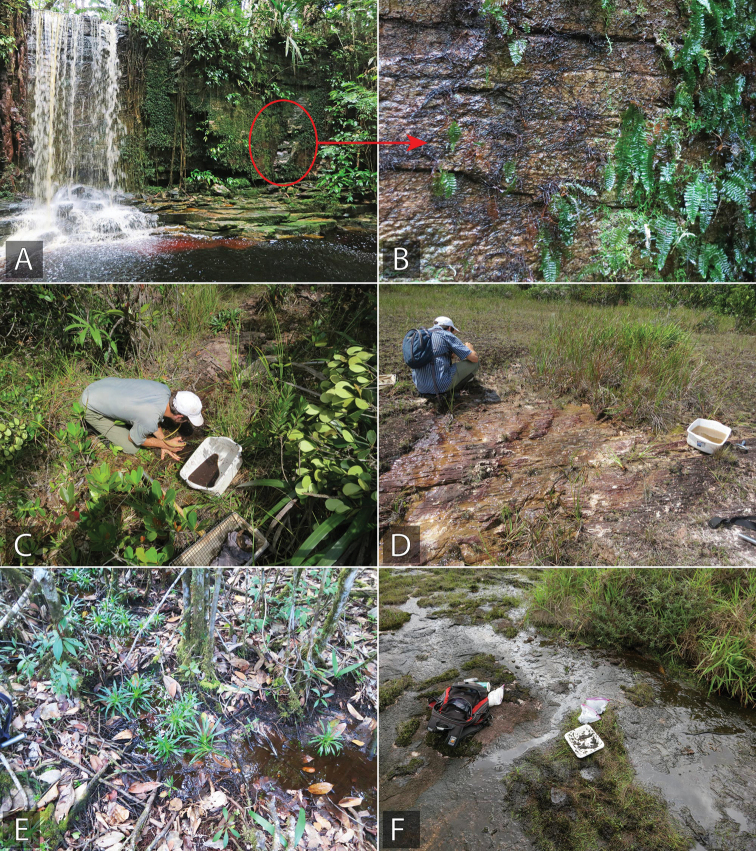
Habitat of *Tobochares* in Suriname **A, B** habitat for *T. kappel*, Petrominas Falls (collecting event SR13-0813-04C) **C** habitat for *T. microps*, Tafelberg Tepui Summit (collecting event SR13-0820-01B) **D** habitat for *T. kappel* and *T. communis*, seepage along Kappel Airstrip (collecting event SR13-0824-02B) **E** habitat for *T. microps*, Tafelberg Tepui Summit (collecting event SR13-0815-01A) **F** habitat for *T. akoerio* and *T. communis* Grensgebergte Mountains (collecting event SR12-0312-01A).

Figs 2H , 3A, D , 6A–C , 11H , 13 , 16D, F

Tobochares 1B: Short et al. (2021).

##### Type material examined.

***Holotype* (male)**: “Suriname: Sipaliwini District: 4°40.432'N, 56°11.079'W; 86 m; Raleighvallen Nature Reserve, base of Voltzberg; flotation of roots and debris from seepage; 17.iii.2016; leg. Short and Girón; SR16-0317-01C” (NZCS). ***Paratypes* (187 exs.): Brazil: Amapá**: 2.60342, -51.33237; 73 m, Calcoene (ca. 42 km NW) on BR-156, Large rock outcrop; 21.vii.2018, leg. Short; Floating rootmats/dirt from seepage, BR18-0721-02B (1, SEMC including DNA voucher SLE1566); 3.87281, -51.78939; 10 m, Oiaqpoque (ca. 5.5 km NE), balneario, 18.vii.2018; leg. Short; seepage area, BR18-0718-01B (1, SEMC, DNA voucher SLE1568). **Roraima**: Amajari Municipality, Serra do Tepequém, Igarape Preto Negro, Cachoeira Leje Preta, 3 36.381'N, 61 42.878'W, 618 m, 14.i.2018, leg. Short and Benetti, BR18-0114-04B (1, SEMC, DNA voucher SLE1494); Caroebe Municipality, Reservoir by Usina de Jatapú, 0.872953, -59.282170, 185 m, 17.i.2018, large wall seep with algae, leg. Short, Benetti, and Santana, BR18-0117-01A (1, INPA, DNA voucher SLE1503). **Guyana: Region VIII**: “5°10.514'N, 59°28.970'W; 440 m; Kaieteur National Park; rock savanna, large seepage area; 16.iii.2014; leg. A. Short; GY14-0316-01C” (51, CBDG, SEMC); same, except “flotation of wet leaves and roots; GY14-0316-01B” (SEMC, 6); same, except “seepage on granite with some vegetation; 21.iii.2014; leg. Short, Salisbury, La Cruz; GY14-0321-02A” (9, SEMC including DNA voucher SLE1022). **Suriname: Sipaliwini District**: “2.46554°N, 55.7700°W; 800 m; Camp 2, Grensgebergte Rock; rock seepages; 12.iii.2012; leg. A. Short; SR12-0312-01A” (23, NZCS, SEMC including DNA Voucher SLE481); “4°40.966'N, 56°11.262'W; 96 m; Raleighvallen Nature Reserve, plateau below Voltzberg; seepage; 28.vii.2012; leg. Short, Maier, McIntosh; SR12-0728-01B” (12, SEMC); same, except “SR12-0728-01C” (6, SEMC); same, except “small seep at margin of plateau; 15.iii.2016; leg. A. Short; SR16-0315-03B” (2, SEMC); “3°47.479'N, 56°8.968'W; 320 m; CSNR: near Kappel airstrip; temporary rivulets in tire tracks; 13.viii.2013; leg. Short, Bloom, Kadosoe; SR13-0813-02A” (1, SEMC); same, except “pond in forest on trail to Tafelberg; 13.viii.2013; leg. Short and Bloom; SR13-0813-05A+B” (1, SEMC); CSNR: Tafelberg Summit, nr. Caiman Creek Camp, leg. Short & Bloom, 19.viii.2013, large seepage area, SR13-0819-01A (1, SEMC, DNA voucher 1047); “3°47.479'N, 56°8.968'W; 320 m; CSNR: near Kappel airstrip; seepage flowing into canal/ ditch on S side of airstrip; 24.viii.2013; leg. Short and Bloom; SR13-0824-02A” (1, SEMC); same, except “SR13-0824-02B” (19, SEMC); “4°40.432'N, 56°11.079'W; 86 m; Raleighvallen Nature Reserve, base of Voltzberg; seepage spot on side of rock; 16.iii.2016; leg. A. Short; SR16-0316-01A” (1, SEMC); same data as holotype (15, SEMC); “2°00.240'N, 55°58.259'W; 374 m; Sipaliwini Savanna Nature Reserve; 4-Brothers mountains; seeps on granite; with algae; 30.iii.2017; leg. Short and Baca; SR17-0330-04A” (6, SEMC); same, except “seeps on granite; by flotation; SR17-0330-04B” (11, SEMC). **Venezuela: Bolívar**: “6°04'54.7"N, 61°23'52.7"W; 509 m; along La Escalera, Highway 10; rock seepages; 14.vii.2010; leg. Short, Téllez, Arias; VZ10-0714-01B” (3, SEMC including DNA Voucher SLE1026); “6°35.617'N, 66°49.238'W; 80 m; Los Pijiguaos; morichal/rock outcrop; 16.ix.2007; leg. A. Short, M. García, L. Joly; AS-07-015” (7, MIZA, SEMC); same, except “seeps and stream at night; 9.vii.2010; leg. Short and Téllez; VZ10-0709-03A” (2, SEMC including DNA voucher SLE1036); “5°40'24.8"N, 61°24'11.3"W; 1330 m; unnamed river; small side stream; 2.viii.2008; leg. Short, García, Joly; AS-08-066” (3, SEMC); “6°57.904'N, 66°36.392'W; 51 m; outcrop ca. 15 Km NE of Los Pijiguaos; detritus flotation; 9.vii.2010; leg. Short and Téllez; VZ10-0709-01B” (4, SEMC); 7°29'47.3"N, 65°51'44.8"W; 45 m; 2 km E of Río Cuchivero; rock outcrop seeps; 6.viii.2008; leg. Short, Téllez, Arias; AS-08-075 (1, SEMC, DNA voucher SLE1031).

##### Differential diagnosis.

By its elytral punctation with all punctures about the same size and degree of impression, *T. communis* is similar to *T. anthonyae* and *T. microps*. From *T. microps*, *T. communis* can be easily distinguished by the relative size of their eyes: in *T. communis* the eyes are separated by a distance 4.5 × larger than the largest diameter of the eye in dorsal view (Fig. 2H), whereas in *T. microps* the eyes are separated by a distance 6 × larger than the largest diameter of the eye in dorsal view (Fig. 2I). In *T. communis* the dorsal punctation is sharper, with all punctures seemingly longitudinally aligned, not forming grooves (Fig. 6A), whereas in *T. anthonyae* the dorsal punctation is relatively shallow, with serial punctures aligned in rows and slightly impressed, forming shallow longitudinal grooves (Fig. 7A).

##### Description.

***Size and form***: Body length 1.8–2.0 mm. Body elongate oval, somewhat parallel sided, moderately convex (Fig. 6A, B). ***Color and punctation***: Dorsal and ventral surfaces of body dark brown, with anterior and lateral margins of prothorax, and outer margins of elytra slightly paler (Fig. 6A); mouthparts yellow (especially maxillary palps) to orange; antennae yellowish brown; legs orange brown with paler (yellow) tarsi (Fig. 6C). Ground punctation on head, pronotum and elytra sharply marked. ***Head***: Eyes in dorsal view with anterior margin oblique (anteriorly directed), and outer margins slightly bulging from outline of head (Fig. 2H); in lateral view, eyes not emarginate (e.g., Fig. 2E). ***Thorax***: Elytra with all kinds of punctures similar in size and degree of impression, all seemingly longitudinally aligned and uniformly distributed, not forming grooves (Fig. 6A). Metafemora mostly glabrous on anterior face (Fig. 6C). Elevation of mesoventrite forming a longitudinal bulge (Fig. 3D). Metaventrite with distinct median, longitudinal, narrow glabrous area extending along posterior half (Fig. 3D). ***Abdomen***: Abdominal ventrites uniformly and very densely pubescent (Fig. 6C). Aedeagus (Fig. 11H). Basal piece nearly 0.4 × the length of a paramere; greatest width of a paramere nearly 0.6 × greatest width of median lobe; outer margins of parameres nearly straight along basal 2/3, then curved inwards; apex of paramere rounded; median lobe roughly sagittate, narrow along apical third, rounded at apex; gonopore situated nearly at midlength of median lobe.

##### Etymology.

Named with the Latin word *communis* meaning common, highlighting the abundance and wide distribution of the species, which is the most commonly found.

##### Distribution.

This species is the most widely distributed of all known *Tobochares*, occurring from the northwest margin of the Guiana Shield in Venezuela all the way to its eastern edge in the state of Amapá, Brazil (Fig. 12).

##### Life history.

This species is strongly associated with seepage habitats on exposed granite. Many of the seepages on which this species has been collected are seasonal, although others are adjacent to permanent streams. See Fig. 16D, F.

#### Tobochares kappel sp. nov.

Taxon classificationAnimaliaColeopteraHydrophilidae

070C7F3C-DA6D-5961-8BFD-6D730015238A

http://zoobank.org/C8875675-BBC9-4121-A77C-8744B1CF31E3

Figs 9A–C , 11L , 13 , 16A, B, D

##### Type material examined.

***Holotype* (male)**: “Suriname: Sipaliwini District: 3°47.479'N, 56°8.968'W; 320 m; CSNR: near Kappel airstrip, wet rocks on sides of waterfall; 13.viii.2013; leg. Short and Bloom; SR13-0813-04B” (NZCS). ***Paratypes* (53 exs.): Suriname: Sipaliwini District**: same data as holotype (49, NZCS, SEMC); same, except “Petromina Falls; leg. Short, Bloom, Kadosoe; SR13-0813-04C” (4, SEMC).

##### Differential diagnosis.

*Tobochares kappel* can be recognized by its strongly convex body in lateral view, accompanied by elytral punctation uniform in size and degree of impression, not impressed to form elytral striae; the serial punctures are seemingly aligned in rows and the interserial punctures are somewhat irregularly distributed and moderately dense (in one or two rows, Fig. 9A, B). The general habitus and punctation of *T. kappel* are similar to those of *T. arawak*, *T. canaima*, and *T. kolokoe*. In *T. arawak* and *T. canaima* the interserial punctures are highly dense (forming two or three irregular rows, Fig. 3I). In *T. kappel* the coloration of pronotum and elytra gradually becomes paler (orange) towards the outer margins (Fig. 9A, B), and the legs are orange in color (Fig. 9C), whereas in *T. kolokoe* only the anterolateral margins of the pronotum are slightly paler and the legs are reddish brown in coloration (Fig. 9D–F). In addition, the serial punctures are equally impressed along the entire surface of the elytra in *T. kappel* (Fig. 9B) whereas in *T. kolokoe* the serial punctures become more impressed along the postero-lateral areas of the elytra (Fig. 9E).

##### Description.

***Size and form***: Body length 1.6–1.9 mm. Body elongate oval, strongly convex (Fig. 9A, B). ***Color and punctation***: Dorsal and ventral surfaces of body dark brown, prothorax (especially its lateral areas) and outer margins of elytra slightly paler (Fig. 9A, B); mouthparts yellow (especially maxillary palps); antennae yellowish brown; legs orange brown with paler (yellow) tarsi (Fig. 9C). Ground punctation on head, pronotum and elytra moderately marked. ***Head***: Eyes in dorsal view with anterior margin oblique (anteriorly directed; e.g., Fig. 2D), and outer margins slightly bulging from outline of head; in lateral view, eyes not emarginate (e.g., Fig. 2E). ***Thorax***: Elytral punctation uniform in size and degree of impression, not impressed to form elytral striae; serial punctures seemingly aligned in rows; interserial punctures somewhat irregularly distributed in one or two rows (Fig. 9A). Metafemora mostly glabrous on anterior face (Fig. 9C). Elevation of mesoventrite forming a very low transverse carina (Fig. 9C). Metaventrite with distinct median, longitudinal, narrow glabrous area extending along posterior half (Fig. 9C). ***Abdomen***: Abdominal ventrites uniformly and very densely pubescent (Fig. 9C). Aedeagus (Fig. 11L). Basal piece nearly 0.5 × the length of a paramere; greatest width of a paramere nearly 0.5 × greatest width of median lobe; outer margins of parameres weakly convex; apex of paramere narrowly rounded; median lobe roughly sagittate, pinched and rounded at apex; gonopore situated nearly at midlength of median lobe.

##### Etymology.

Noun in apposition. Named after the Kappel airstrip, the locality where the species has been collected.

##### Distribution.

Known from two very closely situated localities adjacent to Kappel Airstrip, at the foot to Tafelberg Tepui in Suriname. See Fig. 13.

##### Life history.

Series were collected from two seepage habitats: one vertical seepage on sandstone adjacent to a large waterfall, and the second from a small mostly horizontal seepage on sandstone seeping into an adjacent stream. See Fig. 16A, B, D.

#### Tobochares kolokoe sp. nov.

Taxon classificationAnimaliaColeopteraHydrophilidae

E49F0133-1C21-5F70-A43A-6DE50639C97D

http://zoobank.org/A96013B8-7016-404F-9490-1765F74F737B

Figs 3H , 9D–F , 13

##### Type material examined.

***Holotype* (female)**: “Suriname: Sipaliwini District: CSNR: Tafelberg Summit, Arrowhead Basin; flotation of wet moss on rocks; 20.viii.2013; leg. Short and Bloom; SR13-0820-04A” (NZCS).

##### Differential diagnosis.

*Tobochares kolokoe* can be recognized by its strongly convex body in lateral view (Fig. 9E), accompanied by elytral punctation uniform in size and degree of impression, not impressed to form elytral striae; the serial punctures are seemingly aligned in rows and the interserial punctures are somewhat irregularly distributed and moderately dense (in one or two rows, e.g., Fig. 3H). The general habitus and punctation of *T. kolokoe* are similar to those of *T. arawak*, *T. canaima*, and *T. kappel*. In *T. arawak* and *T. canaima* the interserial punctures are highly dense (forming two or three irregular rows, Fig. 3I). In *T. kolokoe* only the anterolateral margins of the pronotum are slightly paler than the general coloration of the pronotum and the legs are reddish brown in coloration (Fig. 9E, F), whereas in *T. kappel* the coloration of pronotum and elytra gradually becomes paler (orange) towards the outer margins (Fig. 9B), and the legs are orange in color (Fig. 9C). In addition, the serial punctures become more impressed along the postero-lateral areas of the elytra in *T. kolokoe* (Fig. 9E), whereas in *T. kappel* the serial punctures are equally impressed along the entire surface of the elytra (Fig. 9B).

##### Description.

***Size and form***: Body length 1.9 mm. Body elongate oval, strongly convex (Fig. 9E). ***Color and punctation***: Dorsal and ventral surfaces of body dark brown, with prothorax (especially its anterolateral margins) slightly paler (Fig. 9D, E); mouthparts yellow (especially maxillary palps) to orange brown; antennae yellowish brown; legs orange brown with paler (yellow) tarsi (Fig. 9F). Ground punctation on head, pronotum and elytra moderately marked. ***Head***: Eyes in dorsal view with anterior margin oblique (anteriorly directed; e.g., Fig. 2D), and outer margins slightly bulging from outline of head; in lateral view, eyes not emarginate (e.g., Fig. 2E). ***Thorax***: Elytral punctation uniform in size and degree of impression, not impressed to form elytral striae (Fig. 9D); serial punctures seemingly aligned in rows; interserial punctures somewhat irregularly distributed in one or two rows (e.g., Fig. 3H). Metafemora mostly glabrous on anterior face (Fig. 9F). Elevation of mesoventrite forming a very low transverse carina (Fig. 9F). Metaventrite with distinct median, longitudinal, narrow glabrous area extending along posterior half (Fig. 9F). ***Abdomen***: Abdominal ventrites uniformly and very densely pubescent (Fig. 9F).

##### Etymology.

Noun in apposition. Named with the Surinamese word *kolokoe* meaning lucky, as this species is known from a single female specimen.

##### Distribution.

This species is only known from the summit of Tafelberg Tepui, a low-elevation sandstone massif in the center of Suriname (Fig. 13).

##### Life history.

The lone specimen of this species was collected by floating saturated moss that was growing on rocks by a waterfall. The males of this species remain unknown.

#### Tobochares microps sp. nov.

Taxon classificationAnimaliaColeopteraHydrophilidae

73BC8FFD-76D0-5FB5-B78E-4FBB26389077

http://zoobank.org/80D946EC-D4CD-48E8-982A-ECE0059D927E

Figs 2I , 3K , 6D–F , 11I , 13 , 16C

Tobochares 2A: Short et al. (2021).

##### Type material examined.

***Holotype* (male)**: “Suriname: Sipaliwini District/ N3 53.359' W56 10.052', 879m/ CSNR: Tafelberg Summit, near/South Rim, small seepage area/leg. Short and Bloom; 20.viii.2013/SR13-0820-01B” (NZCS). ***Paratypes* (43 exs.): Suriname: Sipaliwini District**: Same data as holotype (38, SEMC including DNA voucher SLE1041); “3°55.600'N, 56°11.300'W; 600 m; CSNR: Tafelberg Summit, near Augustus Creek Camp; muddy stream pools; 15.viii.2013; leg. Short and Bloom; SR13-0815-01A” (1, SEMC DNA voucher SLE1051); same, except “pool in rock; SR13-0820-01C” (2, SEMC including DNA voucher SLE 1043); CSNR: Tafelberg Summit, Arrowhead Basin; flotation of wet moss on rocks; 20.viii.2013; leg. Short and Bloom; SR13-0820-04A (2, SEMC including DNA vouchers SLE1038 and SLE1040).

##### Differential diagnosis.

*Tobochares microps* is unique among *Tobochares* species by the reduced size of the eyes, which are separated by a distance 6 × larger than the largest diameter of the eye in dorsal view (Fig. 2I; the ventral face of the eye is only slightly wider than the antennal club, Fig. 6F), whereas in all other species in the genus the eyes are separated by a distance approximately 4.5 × larger than the largest diameter of the eye in dorsal view (e.g., Fig. 2H; the ventral face of the eye is nearly twice as wide as antennal club; e.g., Fig. 6C). *Tobochares microps* is polymorphic for hindwings, with individuals exhibiting either full size or brachypterous wing forms (e.g., Fig. 3K), a condition so far unique in *Tobochares*. By its elytral punctation with all punctures about the same size and degree of impression (Fig. 6D), *T. microps* is similar to *T. communis* and *T. anthonyae*. Besides the shape of the eyes, *T. microps*, can be distinguished from *T. communis* by its relatively shallower punctation and smaller size (compare Fig. 6D to Fig. 6A). From *T. anthonyae*, in which the dorsal punctation is also relatively shallow (Fig. 7A), *T. microps* can be recognized by the serial punctures seemingly longitudinally aligned and uniformly distributed, not impressed to form grooves (serial punctures clearly aligned in rows and slightly impressed, forming shallow longitudinal grooves in *T. anthonyae*; Fig. 7A).

##### Description.

***Size and form***: Body length 1.6–1.7 mm. Body elongate oval, moderately convex (Fig. 6E). ***Color and punctation***: Dorsal and ventral surfaces of body dark brown, with anterior and lateral margins of prothorax slightly paler (Fig. 6D, E); mouthparts yellow (especially maxillary palps) to orange; antennae yellowish brown; legs orange brown with paler (yellow) tarsi (Fig. 6F). Ground punctation on head, pronotum and elytra moderately marked. ***Head***: Eyes in dorsal view with anterior margin slightly oblique (anteriorly directed), and outer margins nearly half the greatest length of eye, slightly bulging from outline of head (Fig. 2I); in lateral view, eyes not anteriorly emarginate (e.g., Fig. 2E). ***Thorax***: Elytra with all punctures about the same size and degree of impression, all seemingly aligned in rows and uniformly distributed, not forming grooves (Fig. 6D). Metafemora mostly glabrous on anterior face (Fig. 6F). Elevation of mesoventrite forming a broad bulge (Fig. 6F). Metaventrite with distinct median, longitudinal, narrow glabrous area extending along posterior half (Fig. 6F). ***Abdomen***: Abdominal ventrites uniformly and very densely pubescent. Aedeagus (Fig. 11I). Basal piece nearly 0.4 × the length of a paramere; greatest width of a paramere nearly 0.5 × greatest width of median lobe; outer margins of parameres uniformly weakly convex; apex of paramere rounded; median lobe roughly sagittate, narrow along apical third, rounded at apex; gonopore situated basad of midlength of median lobe.

##### Etymology.

Named with the combination of the Latin words *micro*, meaning small, and *ops*, meaning eyes, highlighting the small eyes of the members of the species.

##### Distribution.

This species is only known from the summit of Tafelberg Tepui, a low-elevation sandstone massif in the center of Suriname (Fig. 13).

##### Life history.

Most specimens of this species were collected in seepage habitats by directly floating them out of saturated moss that was on the rock. Several specimens were collected in shallow pools on rock that were adjacent to seepages or streams. See Fig. 16C.

#### Tobochares pemon sp. nov.

Taxon classificationAnimaliaColeopteraHydrophilidae

94F0E5CD-E268-5078-952A-6F7AD3EA1C0B

http://zoobank.org/B5050768-C428-4830-9DCC-617766830551

Figs 2G , 3F , 7G–I , 11E , 13

##### Type material examined.

***Holotype* (male)**: “Venezuela: Bolívar: 5°51'N, 62°33'W; 1700 m; Auyan-tepui; Intercept trap; 7–14.ii.1994; leg. J.L. García, A. Chacón” (MIZA). ***Paratypes* (6 exs.)**: Same data as holotype (6, MIZA, SEMC).

##### Differential diagnosis.

The general orange coloration with dark head of *T. pemon* is quite distinct among *Tobochares*, and particularly among members of the *communis* group. In addition, its elytral punctation is relatively unique, including all kinds of punctures being similar in size and degree of impression, with serial punctures aligned in rows, but not forming grooves, and with interserial punctures somewhat irregularly distributed in a single row (Figs 3F, 7G, H). In addition, the median lobe of the aedeagus of *T. pemon* is unique, as it is uniformly broad throughout and apically broadly emarginate (Fig. 11E); the median lobe in other species typically narrows towards the apex and is usually rounded, except for *T. luteomargo*, which has an emarginated median lobe, but in this case the emargination is deep and very narrow (Fig. 11D).

##### Description.

***Size and form***: Body length 1.7–1.8 mm. Body elongate oval, moderately convex (Fig. 7H). ***Color and punctation***: Dorsal surfaces of body orange brown, with lateral margins of prothorax slightly paler (Fig. 7G, H); ventral surfaces of body (except prosternum) dark brown; mouthparts orange brown; antennae light brown; legs, including tarsi orange brown (Fig. 7I). Ground punctation on head, pronotum and elytra rather shallowly marked. ***Head***: Eyes in dorsal view with anterior margin slightly oblique (anteriorly directed; Fig. 2D); in lateral view, eyes not anteriorly emarginate (e.g., Fig. 2E). ***Thorax***: Elytra with all kinds of punctures similar in size and degree of impression; serial punctures aligned in rows, not forming grooves; interserial punctures somewhat irregularly distributed in a single row (Fig. 3F). Metafemora mostly glabrous on anterior face (Fig. 7I). Elevation of mesoventrite forming a low transverse carina (Fig. 7I). Metaventrite with distinct median, longitudinal, narrow glabrous area extending along posterior half (Fig. 7I). ***Abdomen***: Abdominal ventrites uniformly and very densely pubescent (Fig. 7I). Aedeagus (Fig. 11E) with basal piece nearly 0.6 × the length of a paramere; greatest width of a paramere nearly 0.7 × greatest width of median lobe; outer margins of parameres straight and slightly converging along basal 2/5, then uniformly and widely convex; apex of paramere rounded; median lobe roughly rectangular, with wide and short medial emargination at apex; gonopore situated at apical fourth of median lobe.

##### Etymology.

Noun in apposition. Named after the Pemon, an indigenous tribe that inhabits La Gran Sabana region in Venezuela, where Auyan Tepui is located.

##### Distribution.

This species is known from the famous Auyan-tepui, which is also home to Angel Falls, the highest waterfall in the world. Collected at an elevation of 1700 m, this species is one of the relatively few water beetle taxa known from the “Pantepui Province”, which comprises areas of the Guiana Shield which are greater than 1500 m in elevation (Fig. 13).

##### Life history.

The only known series was collected in a flight intercept trap. Nothing further is known about the habitat or biology of this species.

#### Tobochares romanoae sp. nov.

Taxon classificationAnimaliaColeopteraHydrophilidae

85C62854-4A8D-5C25-B4E9-92A035135F9E

http://zoobank.org/AC8A03A7-CF14-4542-A3D4-AD6F3E4542C2

Figs 2F , 8A–C , 11J , 13 , 14E

##### Type material examined.

***Holotype* (male)**: “Brazil: Roraima: Amajari; 3°36.381'N, 61°42.878'W; 618 m; Serra do Tepequém, Igarape Preto Negro, Cachoeira Leje Preta; at edge of seepage, root mats and moss; 14.i.2018; leg. Short and Benetti; BR18-0114-04B”; DNA voucher SLE1493 (INPA).

##### Differential diagnosis.

*Tobochares romanoae* can be recognized by its elytra with well-defined and moderately impressed rows of serial punctures; the impressed stria I is more strongly impressed along the posterior half of the elytra, resembling a well-developed sutural stria (Fig. 8A); interserial punctures somewhat irregularly distributed (Fig. 8A, B). The general habitus of *T. romanoae* is similar to that of *T. akoerio* and *T. canaima*, especially by the uniformly dark coloration of the pronotum; *T. romanoae* (Fig. 8A) can be distinguished from *T. canaima* by the impressed striae in *T. romanoae* (compare with Fig. 10D); from *T. akoerio* the moderately convex body *T. romanoae* allows its recognition (compare Fig. 8B to 8E). The only known specimen was extracted for DNA, so the dark brown coloration might not reflect the true coloration of the species.

##### Description.

***Size and form***: Body length 2.6 mm. Body elongate oval, moderately convex. ***Color and punctation***: Dorsal and ventral surfaces of body, antennae and legs (except yellowish tarsi) dark brown; mouthparts light brown (Fig. 8A–C). Ground punctation on head, pronotum and elytra moderately marked. ***Head***: Eyes in dorsal view with anterior margin oblique (anteriorly directed), and outer margins bulging from outline of head; in lateral view, eyes not anteriorly emarginate. ***Thorax***: Elytra with all kinds of punctures similar in size and degree of impression; serial punctures aligned in rows, impressed as to form longitudinal grooves (Fig. 8A, B); elytral stria I more strongly impressed along posterior half of elytra, resembling well-developed sutural stria (Fig. 8A); interserial punctures somewhat irregularly distributed (Fig. 8A, B). Metafemora mostly glabrous on anterior face, with hydrofuge pubescence along basal half of antero-dorsal margin (Fig. 8C). Elevation of mesoventrite forming a very low transverse carina (Fig. 8C). Metaventrite with distinct median, longitudinal, narrow glabrous area extending along posterior half (Fig. 8C). ***Abdomen***: Abdominal ventrites uniformly and very densely pubescent (Fig. 8C). Aedeagus (Fig. 11J). Basal piece nearly 0.5 × the length of a paramere; greatest width of a paramere nearly 0.4 × greatest width of median lobe; outer margins of parameres very weakly convex; apex of paramere rounded; median lobe roughly triangular, rounded at apex; gonopore situated at midlength of median lobe.

##### Etymology.

Named after Rosina Romano, Director of Meetings and Membership at the Entomological Society of America (ESA), in recognition of all her hard work and dedication to the society and entomological community.

##### Distribution.

This species is only known from the summit of Serra do Tepequem, a low-elevation sandstone massif in northern Brazil, near the border with Venezuela (Fig. 13).

##### Life history.

The only known specimen was collected by floating rootlets and moss that were present on a rocky seepage formed along the margin of the Igarape (stream) Preto Negro (Fig. 14E).

## Discussion

Members of the genus *Tobochares* are some of the most common water scavenger beetles found in hygropetric seepages in the Guiana Shield region of South America. With this paper, their distribution is expanded not only to the eastern and southern borders of the Guiana Shield region but includes one species in the Brazilian Shield as well. This is a signal that the geographic breadth of the genus is likely much larger, and we have still yet only cracked the surface of our taxonomic knowledge of this lineage.

**Figure 17.**
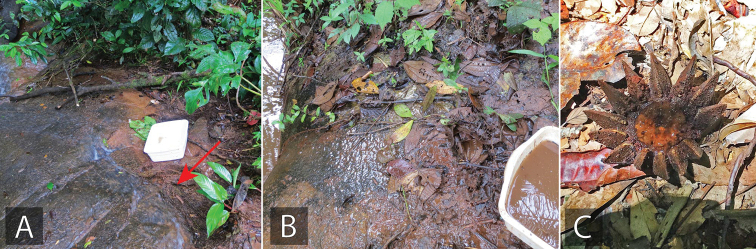
Habitat of *Tobochares fusus*. **A** Brazil, Amapá, near Oiapoque (collecting event BR18-0718-02A), red arrow indicates root mat under which specimens were found **B** Brazil, Amapá, near Oiapoque (collecting event BR18-0718-03C) **C** French Guiana, near Savane Roche Virginie, rotting *Clusia* fruits (collecting event FG20-0310-01D).

Up to now, all species of *Tobochares* for which we have ecological data were associated almost exclusively with hygropetric habitats. These include isolated and seasonal rock seeps on inselbergs to wet rock habitats associated with waterfalls or the wet rock margins of streams and rivers. We report here for the first time the remarkable collection of a *Tobochares* species from both seepage as well as fully terrestrial habitats. We first collected *Tobochares fusus* from several riparian seepage habitats in the Brazilian state of Amapá, just a few kilometers from the border with French Guiana. The longest series was collected on a granite seepage adjacent to a small stream (Fig. 17A), while a second series was collected by washing a root mat that was growing over rock at the margin of a large stream (Fig. 17B). These collections were done in July, a traditionally wet season for the area. To our great surprise, during a separate trip to French Guiana in 2020, we found three specimens of *T. fusus* in rotting *clusia* fruits on dry forest floor, which we confirmed to be genetically identical to those from Brazil (Fig. 1). Within the Acidocerinae, species of the genus *Quadriops* were previously known to be terrestrial (see Girón and Short 2017), and indeed, we also collected a long series of *Q. clusia* Girón & Short, 2017 in the same rotting fruits together with *T. fusus*. The *Clusia* fruits were not near any source of water, and the collection was done in March during a dry period. We did collect in two streams with rocky substrate that were within 1 km of the rotten *Clusia* patch, but while we found many acidocerines, we did not find any *Tobochares* in these aquatic habitats. A nearby inselberg was also completely dry and had no exposed seeps or pools. Taken together, these collections suggest that *Tobochares fusus* is an ecologically vagile species, occurring in both hygropetric as well as fully terrestrial habitats. One possibility could be that the species moves to occupy rotting fruits in the dry season when other seepage habitats are rare or absent. However, more collection effort is required to support this hypothesis.

## Supplementary Material

XML Treatment for Tobochares

XML Treatment for Tobochares sulcatus

XML Treatment for Tobochares benettii

XML Treatment for Tobochares goias

XML Treatment for Tobochares kusad

XML Treatment for Tobochares sipaliwini

XML Treatment for Tobochares striatus

XML Treatment for Tobochares luteomargo

XML Treatment for Tobochares luteomargo

XML Treatment for Tobochares emarginatus

XML Treatment for Tobochares fusus

XML Treatment for Tobochares communis

XML Treatment for Tobochares akoerio

XML Treatment for Tobochares arawak

XML Treatment for Tobochares anthonyae

XML Treatment for Tobochares atures

XML Treatment for Tobochares canaima

XML Treatment for Tobochares communis

XML Treatment for Tobochares kappel

XML Treatment for Tobochares kolokoe

XML Treatment for Tobochares microps

XML Treatment for Tobochares pemon

XML Treatment for Tobochares romanoae
